# Programmable Hydrogels: Frontiers in Dynamic Closed‐Loop Systems, Biomimetic Synergy, and Clinical Translation

**DOI:** 10.1002/advs.202512037

**Published:** 2025-11-14

**Authors:** Guangli Xiang, Bohan Yin, Behzad Shiroud Heidari, George Youssef, Monika Gosecka, Mateusz Gosecki, Fernando G. Torres, Siu Hong Dexter Wong, Jagan Mohan Dodda

**Affiliations:** ^1^ School of Medicine and Pharmacy Ocean University of China Qingdao 266003 China; ^2^ Qingdao Marine Science and Technology Center Qingdao 266237 China; ^3^ School of Biomedical Science The University of Western Australia Nedlands WA 6009 Australia; ^4^ Experimental Mechanics Laboratory Mechanical Engineering Department San Diego State University 5500 Campanile Drive San Diego CA 92182 USA; ^5^ Centre of Molecular and Macromolecular Studies Polish Academy of Sciences Functional Polymers and Polymer Materials Division Sienkiewicza 112 Lodz 90‐363 Poland; ^6^ Department of Mechanical Engineering Pontificia Universidad Catolica del Peru. Av. Universitaria 1801 Lima 15088 Peru; ^7^ New Technologies – Research Centre (NTC) University of West Bohemia Univerzitní 8 Pilsen 301 00 Czech Republic

**Keywords:** adaptive hydrogels, programmable hydrogels, self‐adjustable hydrogels, self‐destructive hydrogels, smart hydrogels

## Abstract

Programmable hydrogels are an emerging class of intelligent materials engineered to respond precisely to specific stimuli, offering tailored functionalities with significant potential for biomedical applications, including drug delivery, tissue engineering, and wound healing. This review comprehensively explores various programmable hydrogels responsive to diverse triggers, including temperature, gene expression, color, shape, and mechanical force. The design and fabrication methods underlying these systems are detailed, highlighting the roles of crosslinkers, adhesion groups, and photosensitive functional groups. Furthermore, the key physical, chemical, and biological properties that govern the performance and functionality of hydrogels are analyzed. The review further examines the mechanisms and recent advancements in self‐executing hydrogels, such as self‐activated, self‐oxygenated, self‐expandable, and self‐powered systems, demonstrating how these innovative designs drive the development of next‐generation programmable hydrogels. The main challenges in hydrogel design, including complexity, reproducibility, and clinical translation, are also addressed. Finally, a perspective on future research directions, highlighting the integration of the latest technologies to realize programmable hydrogels with dynamic closed‐loop responsiveness, bionic synergy, and robust clinical applicability, is offered.

## Graphical abstract

This review summarizes the design principles and key features of programmable hydrogels that respond to multiple stimuli. It then delves into the cutting‐edge mechanisms of self‐executing systems, highlighting their role as the cornerstone of next‐generation programmable hydrogels (NGPHs). Finally, by addressing critical challenges in complexity and translation, the review looks to the future of NGPHs, emphasizing their evolution toward dynamic closed‐loop control, biomimetic intelligence, and successful clinical integration.

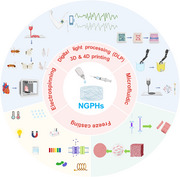

## 1 Introduction

Hydrogels have been widely used in biomedical fields for their biomimetic properties, which closely resemble the natural extracellular matrix (ECM) and provide excellent biocompatibility. However, the advent of programmable hydrogels marks a paradigm shift in material science, transforming these passive, static networks into dynamic, intelligent systems capable of autonomous decision‐making and multifunctional performance. Programmable hydrogels are engineered to exhibit sequential, reversible, and adaptive behaviors responding to environmental cues, making them a cornerstone of next‐generation innovative materials.^[^ ^1^ ^]^ By tailoring their microstructure through covalent and non‐covalent interactions, phase separation, or topological design, programmable hydrogels achieve unprecedented control over their macroscopic properties, enabling them to perform complex tasks with precision and efficiency.^[^ ^2^ ^]^

The development of programmable hydrogels has been propelled by cutting‐edge fabrication technologies such as digital light four‐dimensional (4D) printing,^[^ ^3^ ^]^ lithography,^[^ ^4^ ^]^ and microfluidic spinning,^[^ ^5^ ^]^ realizing intricate, spatially controlled architectures. These advancements, coupled with innovations in dual‐network,^[^ ^6^ ^]^ nano‐composites,^[^ ^7^ ^]^ and topological structures^[^ ^8^ ^]^ have overcome the traditional limitations of hydrogels, such as mechanical fragility and limited functionality.^[^ ^9^, ^10^ ^]^ Programmable hydrogels can be designed to respond to a myriad of stimuli: temperature, pH, light, magnetic fields, and even biological signals, enabling them to adapt their shape, mechanical properties, and functionality in real‐time.^[^ ^11^, ^12^ ^]^ Moreover, integrating microorganisms (e.g., bacteria) and genetically engineered circuits into hydrogel matrices has unlocked entirely new dimensions of functionality, such as self‐glowing^[^ ^13^ ^]^ biosensing,^[^ ^14^, ^15^ ^]^ and autonomous drug delivery, blurring the boundaries between synthetic materials and living systems.

Programmable hydrogels, often referred to as smart or stimuli‐responsive hydrogels, offer a dynamic closed‐loop response that significantly differs from traditional single‐response hydrogel systems.^[^ ^11^ ^]^ Traditional or conventional hydrogels typically exhibit unchangeable swelling properties, an uncontrollable sol–gel transition, lower permeability, and reduced mechanical strength. In contrast, smart hydrogels are designed to respond to specific stimuli on demand, allowing for adaptable functionalities. The core difference lies in the ability of programmable hydrogels to operate within a closed‐loop feedback control system. This means they can sense specific signals in their environment or from biological processes, and then initiate or adjust a specific biofunction, such as releasing a therapeutic agent.^[^ ^16^ ^]^ For instance, smart hydrogels can react to fine fluctuations in their surroundings, including changes in temperature, pH, ionic strength, chemicals, electrical fields, and biological events.^[^ ^11^ ^]^ Furthermore, advanced programmable hydrogels, classified as autonomous biomaterials, can independently adjust their properties and therapeutic delivery in response to changing conditions.^[^ ^16^ ^]^ This dynamic capability allows them to continuously adapt, unlike traditional hydrogels, which typically exhibit a fixed response to a single initial stimulus.^[^ ^11^, ^16^ ^]^ This inherent adaptability in programmable hydrogels enables effective cell adaptation to the matrix and supports related cellular processes through features like degradable structural components or reversible dynamic crosslinks.^[^ ^11^ ^]^

The primary advantage of programmable hydrogels is their ability to integrate multiple autonomous functionalities into a single platform. For instance, hydrogels can generate power from mechanical motion during self‐healing, adapting their shape to fit a dynamic environment and sensing changes in their surroundings to deliver targeted therapies.^[^ ^17^ ^]^ This multifunctional integration is the potential hallmark of next‐generation programmable hydrogels poised to revolutionize fields, ranging from bionic robotics.^[^ ^18^, ^19^ ^]^ and artificial muscles^[^ ^20^, ^21^ ^]^ to wearable electronics and personalized medicine.^[^ ^22^ ^]^

This review delves into the transformative potential of various programmable hydrogels, focusing on their adaptive capabilities, concepts, manufacturing strategies, and mechanisms. We then discuss various evolving hydrogels possessing autonomous properties. Although scattered studies of hydrogels with single autonomous properties, such as self‐healing, self‐powering, and self‐sensing, have already been reported in previous studies,^[^ ^23^, ^24^, ^25^, ^26^ ^]^ a collective reference of programmable properties has not been established as a guide for developing future multifunctional hydrogels. Hence, we review recent advances in programmable hydrogels based on their self‐emanating properties and discuss the design challenges associated with them. We envision that the latest technologies can integrate versatile programmability to formulate the next generation of hydrogels with dynamic closed‐loop, bionic synergy, and clinical orientation as core features.

### 1.1 History and Background

**Figure 1:**
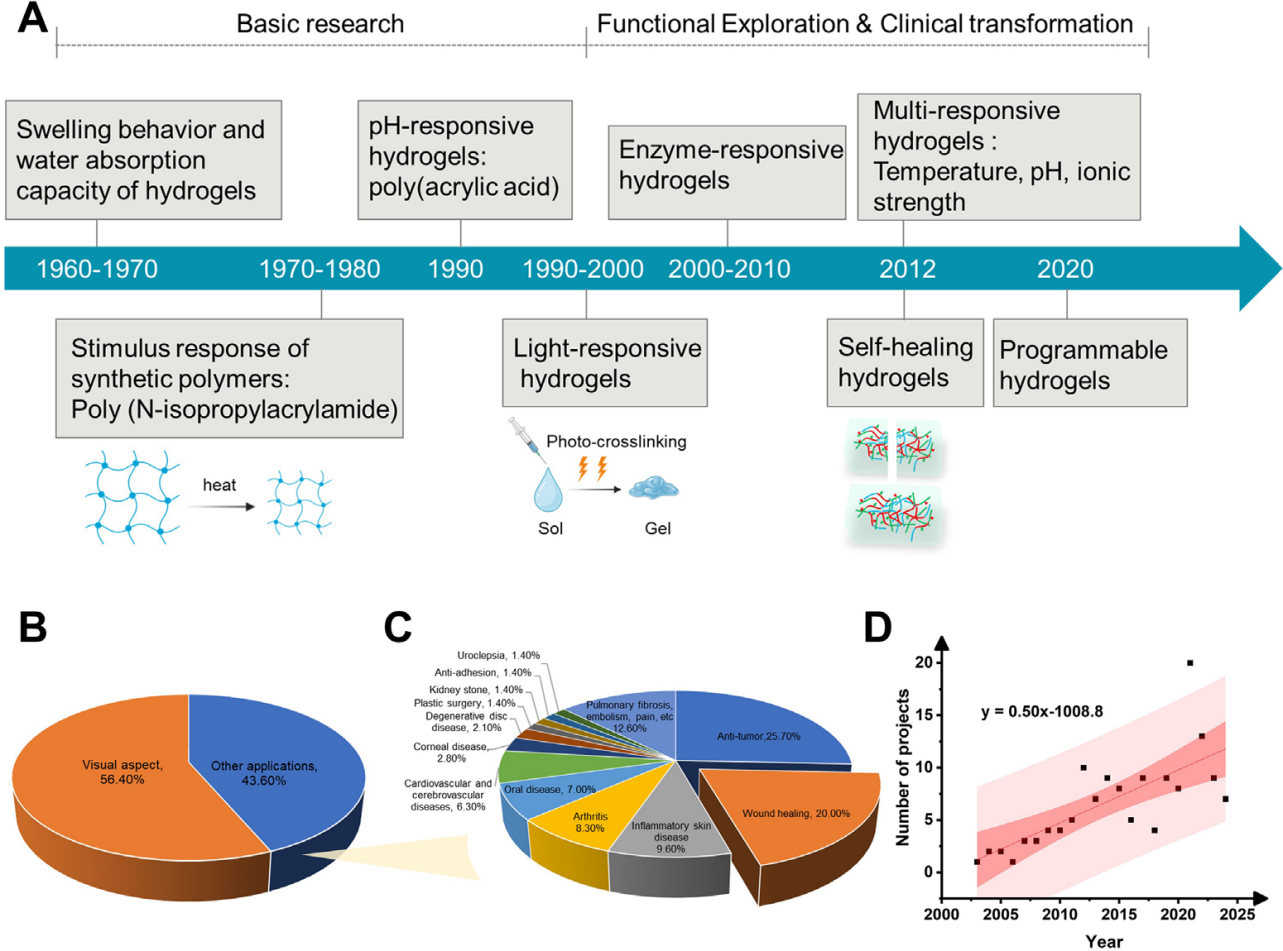
Development history and clinical transformation status of hydrogels. (A) Timeline of progress in hydrogels; (B) Hydrogels approved for clinical trials by the US Clinical Trials Database between 2003 and 2025 (330 in total); (C) Applications of hydrogels (144 in total) entering the clinical program, excluding the use of hydrogels as contact lenses; (D) Hydrogels approved for clinical trials in the United States each year from 2003 to 2025 (excluding the use of hydrogels as contact lenses).

**Figure** **1**A summarizes the technological evolution of hydrogels from basic research to functional exploration and clinical translation, with the era from the 1960s to the 1990s representing the basic research stage. In the 1960s–70s, research focused on the swelling kinetics and absorption properties of hydrogels (such as polyacrylamide systems), laying the theoretical foundation for their use as water absorbent materials.^[^ ^27^ ^]^ Stimulus‐responsive hydrogels emerged in the 1970s–80s, e.g., temperature‐sensitive poly‐n‐isopropylacrylamide (PNIPAM).^[^ ^28^ ^]^ In the 1990s, advances in polymer chemistry and materials science leveraged polyacrylic acid (PAA) and its derivatives, developing pH‐responsive hydrogels.^[^ ^29^ ^]^ Photoreactive hydrogels were developed in the 1990s–2000s by incorporating photoactive molecules or photo‐responsive crosslinkers into networks.^[^ ^30^ ^]^ Since the dawn of the 21st Century, the focus has been on functionalization, enzymatic reaction,^[^ ^31^ ^]^ self‐healing hydrogels,^[^ ^32^, ^33^ ^]^ and multiple stimulus‐responsive hydrogels (such as temperature, pH, light, ionic strength, etc.).^[^ ^34^ ^]^

Drawing on data from the American clinical trial database, we analyze the clinical translation and industrialization of hydrogels from 2003 to 2025, during which 330 hydrogel‐related clinical trials were approved, highlighting significant market demand and application potential. Contact lens materials account for ≈60% of hydrogel applications (Figure 1B), underscoring their maturity in the ophthalmic field. The applications of hydrogels in anti‐tumor (e.g., localized drug delivery) and wound repair represent ≈40% (Figure 1C), while showing a rising trend toward therapeutic materials (Figure 1D). This evolution reflects the transformation of hydrogels from simple swelling carriers to programmable, intelligent systems, laying the groundwork for future multifunctional designs.

**Figure 2:**
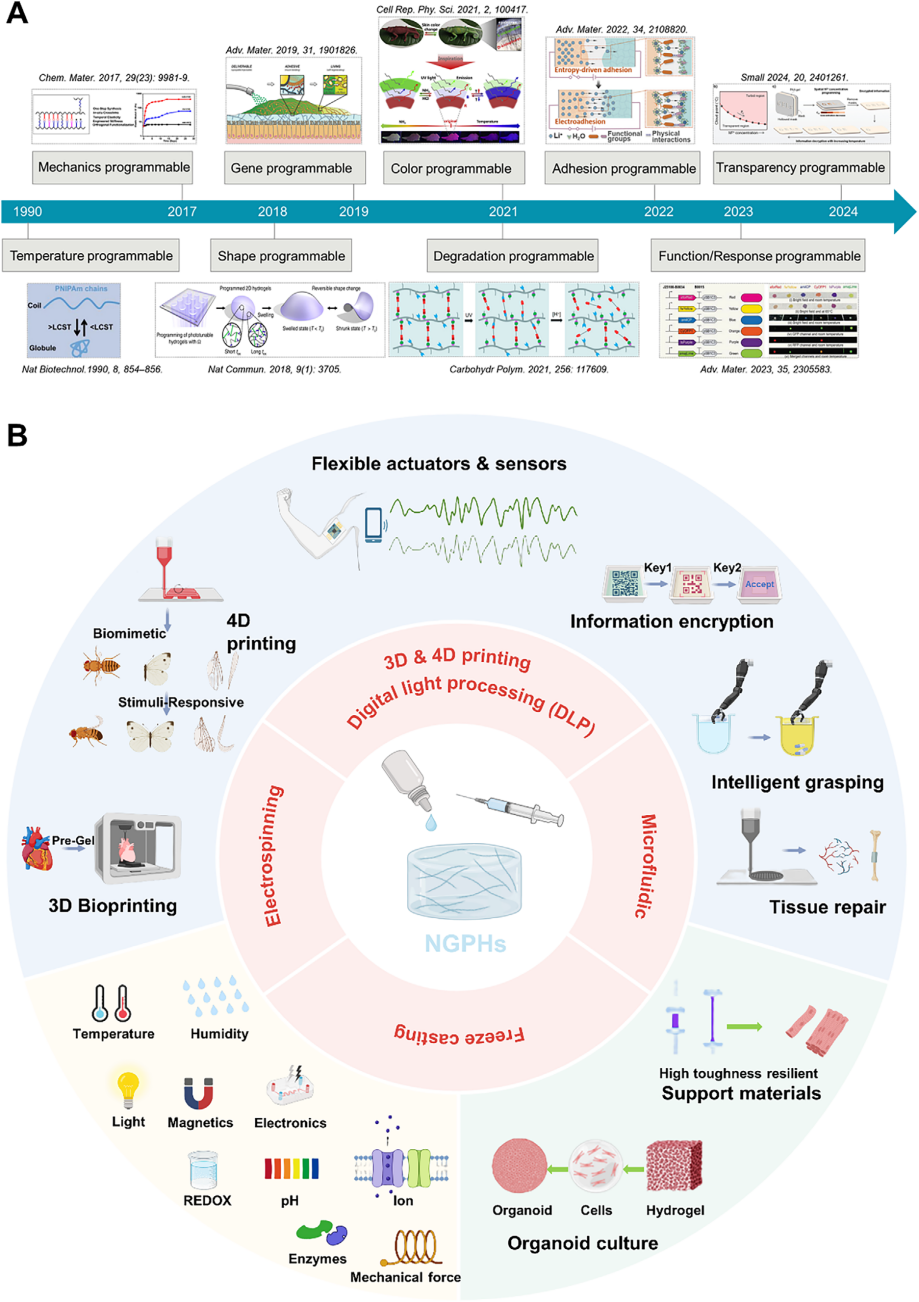
(A) Timeline of advancements in programmable hydrogels. (B) Vision for the Next‐Generation Programmable Hydrogels (NGPHs). These hydrogels will leverage technologies such as digital light processing (DLP), stereolithography (SLA), 3D and 4D printing, microfluidics, electrospinning, and freeze casting. They will feature highly customizable structures, multifunctionality, and intelligent responsiveness, enabling predictable, reversible, or sequential changes in behavior or function in response to preset programs or environmental stimuli. NGPHs hold significant potential for applications in tissue engineering, soft robotics, sensors, shape‐adaptive devices, and beyond. (Created with BioRender.com).

Since the 2020s, diverse programmable hydrogels have emerged^[^ ^35^ ^]^ (**Figure** **2**A). These intelligent materials dynamically adjust their structure, properties, or function in response to external stimuli or pre‐programmed design. With high controllability and multifunctionality, they enable periodic, reversible, or sequential changes in behavior based on predefined “programs” or environmental conditions. While modern programmable hydrogels have advanced beyond traditional stimulus‐response mechanisms, challenges persist, particularly regarding the transport efficiency of signal components (outside‐in) and gel stability. For instance, (i) hydrogels engineered for “controlled degradation” in laboratory settings may behave unpredictably under physiological conditions due to enzymatic degradation, mechanical stress, or other factors, leading to inconsistent release of active components and hindering clinical translation;^[^ ^36^, ^37^, ^38^ ^]^ (ii) conductive hydrogels face significant gaps in long‐term stability (e.g., reduced electrical conductivity after repeated stretching), environmental resilience (e.g., sensitivity to humidity or temperature changes), and industrial applicability.^[^ ^39^ ^]^

Developing next‐generation programmable hydrogels (NGPHs) necessitates trans‐disciplinary knowledge, such as clinical medicine (EMA standards), microelectronics (sensing), fluid dynamics (microfluidics), and employing advanced manufacturing technologies (e.g., digital light processing, stereolithography, 4D printing, microfluidics, electrospinning, and freeze casting). NGPHs feature three core characteristics: dynamic closed‐loop functionality, biomimetic collaboration, and clinical orientation. NGPHs will combine highly customizable structures, integrated multifunctional capabilities, and intelligent information processing, potentially revolutionizing biomedicine and flexible electronics, addressing critical clinical challenges, and meeting stringent industrial demands (Figure 2B).

### 1.2 Types of Programmable Hydrogel

#### 1.2.1 Temperature Programmable Hydrogel

Temperature‐programmable hydrogels respond to temperature changes by altering their physical structure and chemical properties. The response is driven by conformational changes in temperature‐sensitive polymer chains within the gel, resulting in changes to its shape, size, or properties. The programmability of this subclass of hydrogels hinges on precise temperature control, enabling mechanical or functional response at targeted temperatures. Notably, temperature‐programmable hydrogels hold significant promise for tissue engineering.^[^ ^40^ ^]^ Pioneering work began in 1990 with PNIPAM to precisely and reversibly control cell attachment and detachment (Figure 2).^[^ ^41^, ^42^ ^]^ At room temperature, PNIPAM is hydrophilic, but undergoes a reversible phase transition (from hydrophilic to hydrophobic) within the body's temperature range. Below the phase transition temperature, the hydrophilic surface of PNIPAM promotes cell adhesion by interacting with hydrophilic groups on cell surfaces, such as phosphate in the phospholipid bilayer. Above this temperature, the surface becomes hydrophobic, weakening cell adhesion and allowing cell detachment through simple mechanical action or mild external stimuli. This property facilitates applications in cell culture, cell screening, and tissue engineering.

However, traditional temperature‐responsive PNIPAM hydrogels have a narrow temperature window (32–34 °C), which does not match the physiological response, and also have the problem of dehydration caused by temperature changes. This dehydration makes the local areas of the hydrogel with cell‐unfavorable mechanical properties (e.g., high stiffness and small pore size), which may restrict cell‐matrix and cell‐cell interactions for a dynamic microenvironment.^[^ ^43^ ^]^ A previous study modified PNIPAM through copolymerization with polyethylene Glycol (PEG), which created an extra hydrophilic domain to entrap free water molecules to resist dehydration during phase change.^[^ ^44^ ^]^ By changing the PEG molecular weight, the lower critical solution temperature (LCST) of the hydrogel can be increased from 32 °C to above 37 °C, thereby broadening the “temperature window” of traditional PNIPAM to facilitate physiological use.

Additionally, three‐dimensional cell and organoid cultures rely on viscoelastic matrices for mechanical support.^[^ ^45^ ^]^ However, the viscoelasticity and stress relaxation properties of traditional temperature‐programmable hydrogels are hardly optimized for cell growth. Recently, Deoxyribonucleic acid (DNA)‐based hydrogels have demonstrated temperature‐dependent stress relaxation,^[^ ^46^ ^]^ enabling precise control over matrix stress relaxation, crosslinking thermodynamics, kinetics, and degradability through tailored DNA sequence modifications. These gels also support thermally reversible sol–gel transitions and regulated thermal activation, uniformly encapsulating mammalian cells, and promoting the development of diverse cells and organoids.^[^ ^47^ ^]^

In order to meet the requirements of complex tissue construction, a multi‐threshold temperature‐sensitive material is highly desirable for achieving multi‐level responses and precise temperature control. Therefore, developing substitutes for PNIPAM, such as novel intelligent polymers that integrate exceptional durability, multi‐threshold temperature responsiveness, and built‐in optical self‐reporting capabilities (e.g., organic long‐persistent luminescence), represents a major future direction in the advancement of a new generation of temperature‐programmable hydrogels.^[^ ^48^ ^]^

#### 1.2.2 Mechanically Programmable Hydrogel

Mechanically programmable hydrogels sense and convert external stimuli (e.g., light and mechanical force) into physicochemical energy that rearranges/reprogrammes the structural network of the hydrogel.^[^ ^49^ ^]^ These properties offer real‐time regulation of mechanical properties in hydrogels that cannot be achieved by traditional hydrogels.^[^ ^50^, ^51^ ^]^ In 2017, a major breakthrough named “programmable molecular machine” was characterized by orthogonal functionalization (Figure 2).^[^ ^52^ ^]^ In 2020, a nanocomposite hydrogel made of poly(1‐vinylimidazole‐co‐methacrylic acid) and gold nanorods (AuNRs) utilized a post‐photo‐conditioning strategy to achieve photo‐regulated gradient structures for re‐programming its mechanical properties.^[^ ^53^ ^]^ These hydrogels exhibit yielding and forced elastic deformation at room temperature in a glassy state. The gel volume shrinks above the glass transition temperature upon the photothermal effect produced by AuNRs, causing chain segment collapse and the formation of denser intra‐ and inter‐chain hydrogen bonds. The mechanical properties can be recovered after the hydrogel has cooled down to room temperature.

By integrating the cell‐matrix mechanical communication mechanism, the mechanically programmable hydrogel can precisely simulate the biophysical/biochemical properties of the ECM, and can directionally promote cell proliferation by dynamically regulating the mechanical properties of the material (such as stiffness, topological structure), thereby achieving intelligent tissue repair. For instance, in diabetic tissue repair, reduced wound contractility impairs healing.^[^ ^54^ ^]^ Biological materials such as cells or tissues can be a component of hydrogels to biomechanically modulate the biophysical properties of the hydrogel. For instance, mesenchymal stem cells or fibroblasts are mechanosensitive and can exert directional cell traction force on the surrounding matrix. This phenomenon is highly desirable to facilitate diabetic wound contraction based on biomechanics and to speed up wound closure.^[^ ^55^ ^]^ However, optimizing the integration of dynamically programmable hydrogels with biological interfaces is a major breakthrough. Most mechanically responsive hydrogels have insufficient adhesion to tissues, and they are prone to detachment in dynamic physiological environments (such as during wound contraction) and can cause damage to the newly formed tissues when repeated dressing changes are performed.^[^ ^56^ ^]^ Ideally, a hydrogel that permits dynamic cell‐ECM interactions for cell adhesion, cell spreading, and migration can achieve this therapeutic outcome. Recently, a strain‐programmed patch comprising two layers was developed: (i) a non‐adhesive elastomer backing made of hydrophilic polyurethane and (ii) a bioadhesive layer of N‐hydroxysuccinimide (PAA‐NHS ester) and chitosan grafted onto a polyacrylic crosslinked network.^[^ ^57^ ^]^ In vitro studies demonstrated that this patch integrates a dry crosslinking mechanism with a hydration‐based shape memory mechanism, enabling robust and on‐demand detachable adhesion to diabetic wounds. The strain‐programmed patch precisely controls stress concentration and wound contraction at the wound edge, reducing circumferential stress. In mouse, human skin, miniature pig, and humanized mouse models, the patch accelerated diabetic wound healing by applying mechanical contraction, promoting faster epithelial regeneration, angiogenesis, and enrichment of regenerative fibroblast populations. Further, it avoids damage from repeated dressing changes and achieves precise control and active contraction of wet, damaged skin for the first time, offering significant clinical applications. Therefore, a mechanobiology approach can mitigate the adverse effects of devices or pharmacological treatments on wound healing, offering significant translational benefits.

In the future, developing the next generation of mechanically programmable hydrogels that possess both controllable adhesion and multi‐parameter coupling regulation (mechanical and biochemical signals) will be the key to addressing complex pathological microenvironments (such as chronic wounds).^[^ ^58^ ^]^

#### 1.2.3 Shape Programmable Hydrogel

Shape‐programmable hydrogels are intelligent soft materials that undergo complex deformations in response to external stimuli, such as temperature,^[^ ^59^ ^]^ pH,^[^ ^60^, ^61^ ^]^ light, magnetism,^[^ ^62^ ^]^ electricity,^[^ ^63^, ^64^ ^]^ and water.^[^ ^65^ ^]^ Their tissue‐like softness and versatile molecular design for soft actuators, including artificial muscles^[^ ^66^ ^]^ and underwater soft robots.^[^ ^67^ ^]^ The actuation of these hydrogels typically results from changes in osmotic pressure, which drives macroscopic volume changes through swelling/deswelling. However, this osmotic pressure‐driven mass transfer diffusion mechanism inherently limits response speed, often requiring minutes to hours to complete a single actuation cycle. Additionally, the deformation modes of these hydrogels are typically fixed during synthesis, making it challenging to adapt to complex, dynamic real‐world applications. Consequently, developing shape‐programmable hydrogels with rapid response, reversible transformations, and deformations independent of external environmental conditions remains a significant challenge.^[^ ^68^, ^69^ ^]^ In 2018, a breakthrough was achieved in developing a temperature‐responsive 2D hydrogel with locally programmable expansion and shrinkage rates. This innovation enabled the simultaneous printing of multiple custom‐designed 3D structures from a single precursor in a one‐step process within 60 s, offering precise control over the spatiotemporal mechanics (Figure 2).^[^ ^70^ ^]^ Integrating 4D printing, combining 3D printing with stimulus‐responsive materials, has unlocked new possibilities for hydrogels. For example, researchers created a 4D‐printed hydrogel scaffold using amphiphilic and dynamically crosslinked thermoset polyurethane (DTPU) with soft segments consisting of hydrophobic polycaprolactone (PCL) and hydrophilic PEG.^[^ ^65^ ^]^ The low melting point of PCL enables temperature‐triggered shape memory, while the amphiphilic PCL‐PEG network provides programmable deformation and water‐hardening properties. Using multi‐material fused deposition modeling (FDM), DTPU with changing swelling degrees was also printed into 2D patterns. In vitro experiments demonstrated that these 2D sheets rolled into 1D rods around their long axis when submerged in 4 °C water for 10 s. The 1D rod, delivered via a guide wire near a 3.5 mm inner diameter catheter, rapidly unfolded and curled around its short axis upon exposure to water, showcasing pre‐programmed deformation. In vivo studies in rats confirmed the scaffold's ability to transform from 1D to 3D structures. These 4D‐printed hydrogel scaffolds hold promise for applications such as intervertebral disc annulus replacement, supporting soft tissue defects, creating vascular stents, and repairing cartilage.

Other approaches, such as induced structural anisotropy and the incorporation of nanosheets, have also been explored to design shape‐programmable hydrogels, similar to muscle tissue. In 2020, a programmable, reversible shape transformation was achieved using a hydrogel composed of N‐isopropylacrylamide (NIPAM) and stearyl acrylate.^[^ ^71^ ^]^ The structural anisotropy, induced by thermomechanical programming, arose from deformed hydrophobic stearyl domains, which served as a template for the reversible transition of poly Clustered Regularly Interspaced Short Palindromic Repeats and CRISPR‐associated (CRISPR‐Cas) (N‐isopropylacrylamide) chains from a spherical to a coiled conformation. This transient anisotropy could be erased upon cooling, enabling repeatable and reversible shape transformations.

Additionally, macroscopic gel deformation was attained by orienting nanosheets (NS) within hydrogels using a distributed electric field. By incorporating gold nanoparticles (AuNPs) with photothermal properties into a PNIPAM hydrogel, they achieved stable and cyclic deformability through rapid switching of dielectric permittivity and electric repulsion between nanosheets under heat or light exposure.^[^ ^71^ ^]^ When immersed in hot water or irradiated with green light, the hydrogel morphed into a saddle‐like structure, resembling a disk‐like gel with radially arranged nanosheets. Scanning the hydrogel with a 0.8 W/cm^2^ laser beam along its ring at a velocity of 1.05 rad/s induced a traveling buckle, causing the gel to rock back and forth, resulting in rotational and horizontal motion. This localized irradiation enabled asymmetric deformation and centroid shifts in annular hydrogels, with motion controlled by repeated optical scanning. Compared to earlier methods, such as parallel plate electrode‐oriented mask‐assisted photocuring, generating ordered structures using heat or distributed electric fields offers greater customizability of shape‐programmable hydrogel regions and higher preparation efficiency.

#### 1.2.4 Gene Programmable Hydrogel

Gene‐programmable hydrogels can respond to specific genetic instructions or sequences, typically through the complementary hybridization of nucleic acids. Furthermore, integrating gene‐editing technologies, such asthe Clustered Regularly Interspaced Short Palindromic Repeats and CRISPR‐associated (CRISPR‐Cas) system, can achieve precise control over their physical properties (e.g., transparency, stiffness, or shape) or controlled release of cargos.^[^ ^72^ ^]^ Their highly sensitive, selective, and modular construction makes them promising for diagnostic and therapeutic applications. Traditional gene‐programmed hydrogels are prone to non‐specific gene cleavage in complex biological environments, resulting in uncontrolled hydrogel functions or cytotoxicity.^[^ ^73^ ^]^ In contrast, hydrogels based on CRISPR‐Cas have been used as portable, rapid, and quantitative biosensors to distinguish bacterial genotypes,^[^ ^74^ ^]^ detect exosomes,^[^ ^75^ ^]^ and detect cell‐free tumor DNA mutations in vitro.^[^ ^76^ ^]^ This system ascertains the accuracy, efficiency, and spatiotemporal control of gene‐editing applications.

A paramount concern is the potential for off‐target DNA cleavage, where the CRISPR machinery unintentionally alters segments of the host genome.^[^ ^77^ ^]^ These unintended genetic modifications, which can include insertions, deletions, or larger structural variations, carry the risk of leading to adverse health effects, including an increased likelihood of cancer.^[^ ^78^, ^79^, ^80^, ^81^ ^]^ Ensuring genome‐wide specificity is critical, particularly for in vivo applications where the precise selection of edited cells is not feasible.^[^ ^79^ ^]^ Furthermore, the immunogenicity of the Cas proteins, often derived from bacteria, poses a substantial barrier to clinical translation.^[^ ^82^, ^83^, ^84^ ^]^ Many individuals may harbor pre‐existing immune responses to these proteins, which can lead to neutralization of the CRISPR system, resulting in a loss of therapeutic efficacy or even inducing inflammation and tissue damage.^[^ ^84^, ^85^, ^86^ ^]^ Mitigating these immune reactions is crucial for the long‐term success and safety of CRISPR‐based therapeutics. For instance, DNA‐based smart hydrogels utilizing the Cas12a enzyme for programmed nucleic acid cleavage offer high sensitivity, selectivity, and modularity. These hydrogels can provide a suitable microenvironment for cell culture, promoting cell growth and differentiation, or can be used to construct artificial tissues. When customized to interact with specific tissues in the gastrointestinal tract selectively, they pave the way for therapeutic materials with prolonged intestinal residence times.^[^ ^87^ ^]^

Beyond the direct effects of CRISPR, delivery challenges, and the broader biocompatibility of the integrated novel materials add layers of complexity. Improper or off‐target delivery of the CRISPR complex can lead to unintended biological outcomes.^[^ ^86^ ^]^ Moreover, the combination of genetically modified components with new chemical formulations within hydrogels necessitates rigorous assessment of their interactions with physiological tissues to prevent adverse reactions such as allergies, chronic inflammation, or increased susceptibility to infection.^[^ ^88^, ^89^ ^]^ While hydrogels are generally considered biocompatible, the advent of “living biomaterials” containing genetically modified bacteria also introduces serious bacterial safety concerns that must be addressed for broader application.^[^ ^90^, ^91^, ^92^ ^]^

Conventional hydrogels for the mucosal layer, such as the gastrointestinal tract, suffer from long‐term mucosal adhesion and resistance to intestinal peristalsis clearance.^[^ ^87^ ^]^ Recent studies have reported hydrogels that can adapt to biological interfaces and trigger in situ adhesion. For instance, researchers have developed a genetically programmable hydrogel using nanofibers derived from secreted mucosal adhesion proteins produced by engineered non‐pathogenic bacteria (Figure 2).^[^ ^93^ ^]^ The genetically programmable functionality of this hydrogel is demonstrated by its rheological properties, which can be regulated through genetic approaches, along with its ability to specifically adhere to target gastrointestinal tissues and regenerate within days. Furthermore, the hydrogel can be directly obtained from bacterial culture without the need for purification, offering a novel genetically programmable bioactive material platform for long‐term treatment of gastrointestinal wounds. Ensuring the safety and biocompatibility of gene‐programmable hydrogels is imperative in medical applications.^[^ ^94^ ^]^ Advanced gene‐editing techniques, such as enhanced specific Streptococcus pyogenes Cas9 (eSpCas9), high‐fidelity SpCas9 (SpCas9‐HF1), and ultra‐precise Cas9 (hypaCas9) mutants, have been developed to minimize off‐target effects and enhance specificity.^[^ ^95^ ^]^ These advancements provide a robust foundation for improving the safety and biocompatibility of gene‐programmable hydrogels, facilitating their clinical translation.

#### 1.2.5 Color Programmable Hydrogel

Many organisms, such as marine mollusks, butterflies, fish, and spiders, utilize dynamic color changes for adaptive camouflage, concealment, and signaling.^[^ ^96^ ^]^ Hydrogels are ideal candidates for incorporating these biological color modalities as readouts of responsiveness and biosensors. However, traditional color‐programmable hydrogels face three major challenges: stimulus responses rely on chemical methods, leading to insufficient control precision,^[^ ^97^ ^]^ color performance suffers from limited spectrum coverage, inflexible multicolor switching, and poor stability, while system integration is hindered by shortcomings in fabrication processes and interactive capabilities. To address these issues, recent research focus has shifted toward developing clean stimulation methods such as magnetic fields,^[^ ^98^ ^]^ electricity,^[^ ^99^ ^]^ metal ions,^[^ ^100^ ^]^ and heat,^[^ ^101^, ^102^ ^]^ adopting novel chromogenic materials and core‐shell structures to enhance performance, and promoting intelligent and practical applications through innovative technologies like flexible bionic skins and optical writing. For instance, inspired by the natural color‐changing abilities of leopards, researchers in 2021 designed a multi‐light‐source supramolecular hydrogel system (Figure 2).^[^ ^103^ ^]^ They incorporated red Eu3+‐aminopyridine (R) luminescence into the hydrogel core and blue naphthalimide (B) and green perylene tetracarboxylic acid (G) luminescence into its supramolecular shell. By independently controlling the intensity of the G/B luminescent components, its emission color was programmed to shift from red to blue or green, achieving programmable multicolor fluorescence. This hydrogel also demonstrated excellent cyclic color‐changing properties, making it a promising candidate for anti‐counterfeiting applications. In conclusion, color‐programmable hydrogels are valuable for providing real‐time, visual environmental feedback, which is critical for developing intelligent biosensors. Their potential in tissue growth and other biomedical applications warrants further research and development.^[^ ^104^ ^]^

#### 1.2.6 Degradable Programmable Hydrogel

Degradability is a critical factor in optimizing the safety, precision, and administration frequency of hydrogels for biomedical applications. To achieve controlled degradation, responsive molecules or functional groups are incorporated into hydrogel matrices. However, precise modulation of degradation rates remains a significant challenge. Various stimuli, including light, pH, enzymes, and CRISPR‐based systems, have been integrated into the hydrogel to enable programmable control over the crosslinked structure or composition, thereby tailoring its degradation profile.^[^ ^37^, ^105^, ^106^, ^107^ ^]^ The primary advantage of these degradable programmable hydrogels is their tunable degradation rate, which enables precise control over drug release to meet the therapeutic needs of different diseases.

The Diels–Alder (DA) click reaction plays a significant role in developing degradable programmable hydrogels.^[^ ^108^ ^]^ For example, researchers used the DA click reaction to create a semi‐interpenetrating polymer network wound dressing made of hyaluronic acid, polyethylene glycol, and halogen‐free imidazole polyionic liquid (PIL), loaded with deferoxamine (DFO).^[^ ^38^ ^]^ This hydrogel fully degraded in 100 U/mL hyaluronidase at 37 °C within 24 h. During the first 180 min of degradation, the release rate of antimicrobial PILs (45%) was faster than that of the angiogenic agent DFO (34%), enabling progressive hydrogel degradation that supports programmed repair of full‐thickness skin wounds. Similarly, researchers synthesized PEG derivatives with photosensitive o‐nitrobenzyl ester linking groups and terminal maleimide groups, combined with 5‐methylfurfural‐grafted carboxymethyl chitosan derivatives, to form sequentially degradable hydrogels via the DA reaction.^[^ ^37^ ^]^ Under 365 nm ultraviolet light, the photo‐generated o‐nitrosobenzaldehyde product forms pH‐sensitive Schiff base bonds with amino groups, which can be cleaved under acidic conditions to achieve sequential degradation. In contrast, single or non‐cascading stimuli (e.g., continuous UV light and alkaline pH) do not trigger degradation. These hydrogels also exhibit excellent controlled release of rhodamine B, highlighting their broad potential in biomedical applications.^[^ ^109^ ^]^

For diseases requiring frequent drug administration, achieving tissue‐specific localization and controlled release of therapeutics is essential for sustained efficacy. The incorporation of hindered urea bonds (HUB) has been shown to accelerate hydrogel degradation, enabling continuous replenishment of damaged tissues, such as articular cartilage.^[^ ^77^ ^]^ Additionally, researchers developed a novel hydrogel by combining dodecyl‐modified hydroxypropyl methylcellulose (HPMC) with a biodegradable nanosolution of PEG‐PLA containing a target drug.^[^ ^110^ ^]^ This hydrogel reduced the frequency of diabetes drug injections to three times per year, offering significant potential to improve the quality of life for patients with diabetes and obesity.

#### 1.2.7 Adhesive Programmable Hydrogel

**Figure 3:**
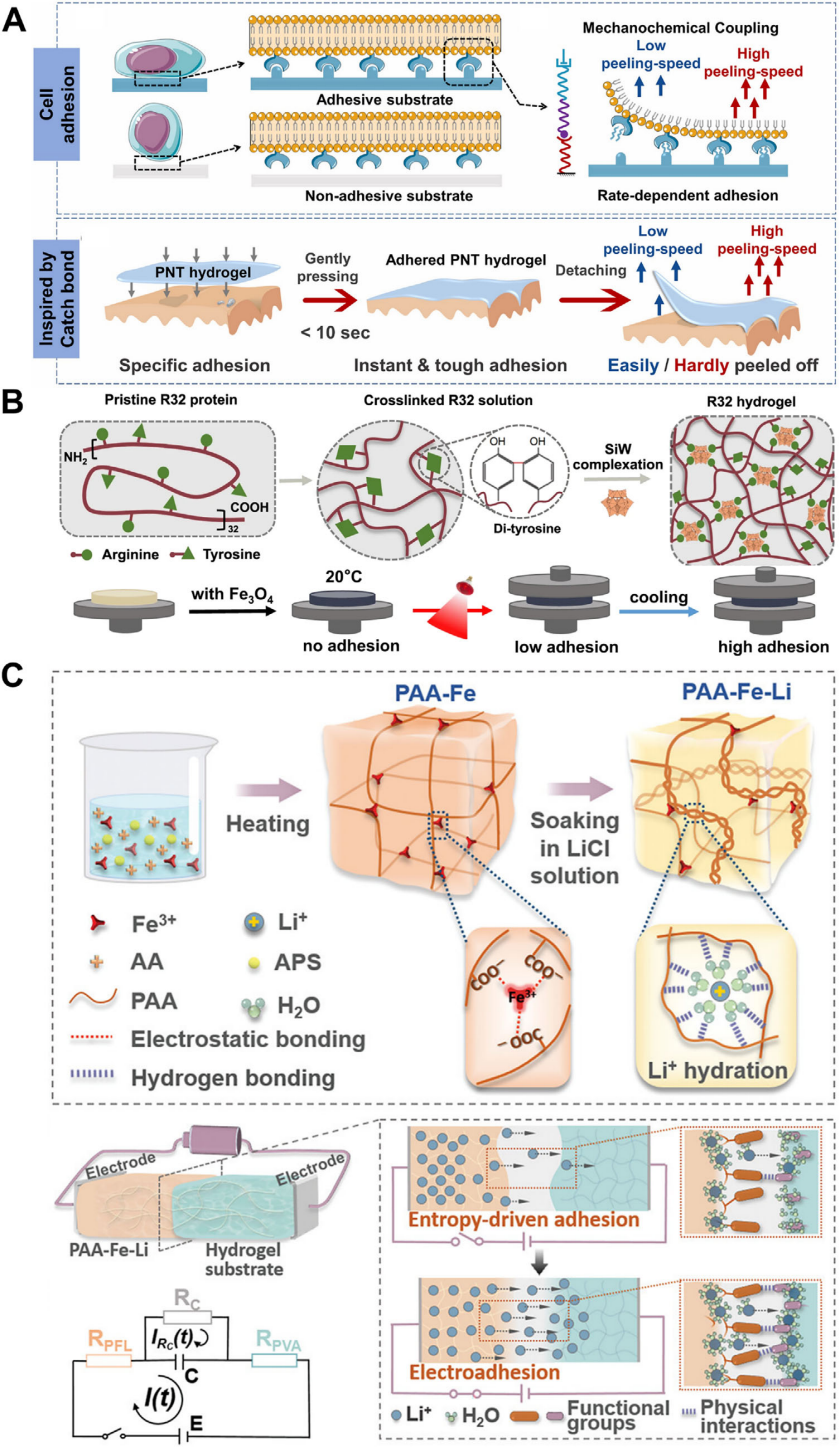
Preparation and application of adhesive programmable hydrogels. (A) Design basis for the catch‐bond inspired hydrogels (PNT hydrogels) demonstrating loading rate‐responsive behavior and specific adhesion. Reproduced with permission.^[^ ^118^ ^]^ Copyright 2022, Elsevier. (B) Proposed design principle of the R32 hydrogels and schematic diagram showing infrared (IR) light‐responsive switching of the hydrogel adhesion. Reproduced with permission^[^ ^117^ ^]^ Copyright 2024, Springer Nature. (C) Design and application of programmable hydrogels with adhesion properties. R_PFL_ and R_PVA_ denote the equivalent series resistances of PAA–Fe–Li and polyvinyl alcohol (PVA) hydrogels. C indicates the effective capacitance of the IAR, and RC represents the effective resistance of the capacitor. E is the electromotive force of the circuit. I(t) and I_RC_(t) denote the corresponding summed and local currents of the hydrogel bonding structures, respectively. Reproduced with permission^[^ ^116^ ^]^ Copyright 2022, Wiley‐VCH.

Adhesion in biological systems, such as cell attachment to the extracellular matrix, is fundamental for maintaining structural integrity and enabling physiological functions.^[^ ^111^, ^112^ ^]^ Alternatively, interfacial adhesion to tissue can be useful for wearable devices or wound dressing. Traditional adhesive hydrogels face challenges, including poor adhesion in wet environments, limited substrate compatibility, and inadequate control over adhesion properties. To address these limitations, hydrogels can be engineered to adhere to diverse substrates through mechanisms such as interface bonding,^[^ ^113^ ^]^ surface modification,^[^ ^114^ ^]^ and topography control.^[^ ^115^ ^]^ Recent advancements have further enhanced hydrogel adhesion through innovative approaches, including electric field‐controlled ion diffusion,^[^ ^116^ ^]^ ordered protein assembly,^[^ ^117^ ^]^ bioinspired reverse locking mechanisms,^[^ ^118^ ^]^ and electrically activated mucosal adhesion.^[^ ^119^ ^]^ These techniques enable the development of programmable adhesive hydrogels with improved performance and versatility for biomedical and technological applications. For example, a catch bond‐inspired smart hydrogel enables repeatable and loading rate‐sensitive specific cell adhesion. The core mechanism mimics the mechanosensitive binding behavior observed in biological systems (e.g., integrin‐ligand interactions), thereby achieving both specific adhesion of mammalian cells and loading rate‐dependent adhesion enhancement (**Figure** **3**A).^[^ ^118^ ^]^ Furthermore, people also utilize the surface network topology structure to control adhesion, dynamic properties, and spatial distribution, demonstrating the adhesion characteristics of programmable hydrogels.^[^ ^120^ ^]^ The mechanism relies on the hydrogel surface network forming supramolecular bonds with varied adhesion behaviors at the gel interface, enabling stable and tunable adhesion dynamics. By combining different topologies, researchers achieved spatially programmable and dynamically adjustable adhesion. This approach successfully integrated multiple facets of adhesion controllability into the hydrogel network, elucidating the roles of polymer chain slippage, fracture, and diffusion in programmable adhesion behavior. A programmable hydrogel adhesion strategy applies to smart wound patches, fluidic channels, drug‐eluting devices, and reconfigurable soft robots. In another study, R32 hydrogel was prepared using dynamic electrostatic interactions between R32 protein and silicotungstic acid.^[^ ^117^ ^]^ This hydrogel exhibits a thermoresponsive property that shows a high Young's modulus and low fracture strain below the threshold temperature (Ts), while shifting to a low Young's modulus and high fracture strain above the Ts. By incorporating Fe_3_O_4_ nanoparticles, soft robots were created that respond to light, heat, and magnetic fields, rapidly switching between soft adhesive and complex non‐adhesive states in response to temperature changes, as well as static interactions between the R32 protein and SiW acid (Figure 3B). The R32 hydrogel shows potential applications such as artificial blood vessel repair and underwater grasping and release, paving the way for underwater robots with on‐demand functionalities.

By utilizing an external electric field to drive the diffusion of ions, the electro‐adsorption phenomenon that can be controlled in real time was also achieved in the hydrogel.^[^ ^116^ ^]^ A saline‐rich hydrogel (PAA‐Fe‐Li) was designed through a two‐step process (Figure 3C). The strong hydration capacity of lithium ions (Li⁺) influenced polymer chain interactions within the hydrogel, affecting interfacial bonding through diffusion and accumulation at the interface (Figure 3C). When the PAA‐Fe‐Li hydrogel contacted a substrate, Li⁺ diffused from the hydrogel to the substrate due to concentration gradients. The entropy‐driven Li⁺ diffusion and accumulation were typically self‐directed and influenced by the hydrogel hydrophilicity, charge, or crosslinking density, reducing bonding efficiency and controllability (Figure 3C). Li⁺ accumulation at the adhesive interface also formed an ionic adhesion zone (IAR) with capacitive characteristics. By applying an external electric field, researchers precisely controlled the spatiotemporal dynamics of Li⁺ diffusion within the IAR, enabling hydrated ion diffusion‐mediated interfacial adhesion. A forward voltage enhanced adhesion, while a reverse voltage facilitated on‐demand detachment (Figure 3C), paving the way for high‐performance adhesive hydrogels with programmable functionalities.^[^ ^116^ ^]^

#### 1.2.8 Function/Response Programmable Hydrogel

The development of biomaterials requires a delicate balance between structural stability, mechanical properties, and functional programmability. Programmable hydrogels, with their dynamic responsiveness, structural adaptability, and customizable functionalities, offer a promising solution to this challenge. These properties enable their widespread application in fields such as soft robotics, biomedicine, and flexible electronics. However, current magnetic soft robots, which primarily utilize elastomer‐based magnetic composites for programmable geometric and magnetization designs, are limited by the minimal biological functionality of elastomer matrices.^[^ ^121^ ^]^ Traditional magnetic hydrogel robots (MHRs) face challenges in constructing heterogeneous structures, achieving precise magnetization programming, and integrating sensing capabilities.

To overcome these limitations, researchers have developed advanced MHRs by creating chemically crosslinked interfaces between magnetic hydrogels and patterned elastomer films. This approach enables programmable shape‐morphing, precise magnetization control, and multimodal drug manipulation. Further integration of microelectronics has facilitated the development of closed‐loop systems that combine physiological sensing, drug delivery, and magnetic actuation, establishing new paradigms for implantable medical platforms and soft robotics.^[^ ^122^ ^]^ Additionally, innovative approaches integrating materials science, synthetic biology, and microfluidics have led to the development of a sheath‐core live hydrogel fiber platform incorporating living bacteria (Figure 2).^[^ ^5^ ^]^ The inclusion of living bacteria imparts programmable functionalities and life‐like capabilities to the hydrogel. By engineering the genetic circuits of the bacteria to express specific pigments and fluorescent proteins, researchers have achieved color regulation and biosensing functionalities.^[^ ^5^ ^]^ This optimized structure‐performance‐function active hydrogel fiber platform provides a novel tool to accelerate the practical application of emerging active material systems.

#### 1.2.9 Transparent Programmable Hydrogel

The structure of hydrogels is intrinsically linked to their functionality, with functional requirements driving innovations in structural design. Transparent programmable hydrogels, in particular, have garnered significant attention due to their ability to integrate programmable attributes such as shape, color, degradability, and responsiveness to external stimuli. The microstructure of hydrogels can be precisely modulated by external stimuli, including temperature, light, pH, solvents, ionic strength, and mechanical forces. Such stimuli induce controlled changes in geometric and optical properties of the hydrogel, such as transparency and fluorescence, enabling tailored functionalities. This adaptability makes hydrogels ideal for developing anti‐counterfeiting systems with embedded encryption capabilities for secure information storage and transfer. Conventional hydrogels typically rely on a single stimulus‐response mechanism, limiting their encryption to a single level of security. To address this limitation and enhance encryption robustness, researchers are developing transient anti‐counterfeiting hydrogels with dual‐ or triple‐level encryption capabilities.^[^ ^123^ ^]^ The core design principle involves integrating two or three functional structures (e.g., fluorophores) into the polymer network, where the initial and final states correspond to specific encryption and decryption messages. For instance, Nonoyama et al. reported a PAA gel integrating with Ca, referred to as PAA/Ca gels, featuring two distinct transitions: a rubber‐to‐glass transition and a transparent‐to‐cloudy transition.^[^ ^124^ ^]^ They proposed a straightforward strategy for transient anti‐counterfeiting by incorporating bivalent metal (M^2^⁺) coordination complexes into the PAA gel, where the cloud temperature (Tc) could be tuned by varying M^2^⁺ concentrations (Figure 2). By leveraging spatially selective diffusion of M^2^⁺ at different concentrations, multiple T_c_ regions within the gel were locally programmed. As the temperature increased or a complexing agent was added, the transparency of these T_c_ regions evolved under natural light, enabling a transient anti‐counterfeiting process. The researchers integrated a complex quick response (QR) code and a numerical sequence into the hydrogel for practical applications. The QR code, programmed on the gel's top surface using a 0.5 M calcium acetate (CaAc_2_) solution, required a password upon scanning with a smartphone. The numerical sequence, formed on the gel's bottom surface using four CaAc_2_ solutions with varying calcium ion (Ca^2^⁺) concentrations, provided the decryption numbers. Entering the correct numbers redirected the user to a designated website, offering novel anti‐counterfeiting hydrogel materials.

## 2 Fabrication of Programmable Hydrogels

**Table** **1** summarizes the preparation strategies and applications of programmable hydrogels, which offer exceptional modularity, customizability, precise drug delivery, and intelligent responsiveness. Advances in materials science and biomedical engineering continue to unlock new techniques and possibilities for designing next‐generation programmable hydrogels. The latter hinges on a deep understanding of material properties and underlying mechanisms. Researchers have created advanced adaptive materials by emulating the complex reaction networks and feedback regulation mechanisms observed in biological systems. These materials exhibit unique spontaneous formation mechanisms and “self” properties. The following sections categorize hydrogels based on their “internal” properties and mechanisms and their “external” adaptive or self‐regulating functionalities. Specifically, we classify them into two groups: (1) self‐assembling, self‐growing, and self‐crosslinking hydrogels, and (2) adaptive, self‐regulating, and self‐adhesive hydrogels, which will be discussed in Sections 2.1 and 2.2, respectively.

**Table 1:** Design strategies and applications of various programmable hydrogels.

Name	Strategy	Application	Refs.
Temperature	Use temperature‐sensitive polymer (PNIPAM), or adjust Lower Critical Solution Temperature (LCST) or Upper Critical Solution Temperature (UCST)	Drug delivery, temperature sensors, tissue engineering	[28, 124]
Mechanical	Change crosslinking density or polymer chain orientation, use shape memory effect polymers or nanomaterials, dual network structure, and dynamic bonding.	Tissue engineering, soft robotics, biosensing	[4, 49]
Shape	Adjust crosslinking density, polymer chain orientation, and use shape memory materials.	Soft robots, smart textiles, adaptive structures	[62, 68, 104]
Gene	Integrate genetically engineered materials to regulate performance through gene expression.	Gene therapy, biosensing, and tissue engineering	[72, 125, 126]
Color	Use photosensitive materials, photonic crystals, or responsive dyes to regulate color by light, pH, or temperature.	Sensors, displays, and anti‐counterfeiting materials	[1, 5, 104]
Degradability	Use degradable crosslinkers or stimulate sensitive (enzyme, REDOX) polymers to regulate the degradation rate.	Drug delivery, tissue engineering, and environmental protection	[37, 127]
Adhesiveness	Introduce adhesion groups (such as dopamine)	Biomedical glue, tissue repair, flexible electronics	[5, 116, 117]
Function/response	Use responsive polymers or regulate material surface chemistry and topological structure.	Targeted delivery, biosensing	[128]
Transparent	Regulate crosslinking density, mix nanomaterials, or use phase change materials.	Smart Windows, optics, and cloaking materials	[124]

### 2.1 Self‐Assembling/Self‐Crosslinking Hydrogels/Self‐Growing

Self‐assembling, self‐growing, and self‐crosslinking hydrogels are defined by their material properties and internal mechanisms, exhibiting spontaneous formation without external intervention.

#### 2.1.1 Self‐Assembly And Self‐Crosslinking Hydrogels

At a macroscopic level, self‐assembling and self‐crosslinking hydrogels share inherent, self‐driven gelation properties but differ in formation mechanisms, characteristics, and applications. Self‐assembled hydrogels are mainly prepared from small molecules,^[^ ^129^ ^]^ biomacromolecules,^[^ ^130^ ^]^ polymers,^[^ ^131^ ^]^ peptides,^[^ ^132^ ^]^ and other substances dependent on non‐covalent interactions, such as electrostatic interaction,^[^ ^133^ ^]^ π‐π stacking,^[^ ^134^ ^]^ van der Waals force,^[^ ^135^ ^]^ hydrophobic interaction,^[^ ^136^ ^]^ and metal coordination bonds.^[^ ^137^ ^]^ In contrast, self‐crosslinking hydrogels rely on crosslinking points or reactive groups within the molecules themselves, which are formed through chemical reactions (e.g., click chemistry,^[^ ^102^ ^]^ enzyme‐catalyzed reactions,^[^ ^138^ ^]^ high‐energy irradiation^[^ ^139^ ^]^) or physical interactions (e.g., hydrogen bonding and electrostatic interactions). For example, in alginate self‐crosslinking hydrogels, alginate dialdehyde (ADA) chemically crosslinks with gelatin through the Schiff base reaction. At the same time, borax forms hydrogen bonds with alginate's hydroxyl groups, facilitating physical self‐crosslinking within the gel.^[^ ^140^ ^]^ Based on their formation mechanisms and network bonding, self‐assembling hydrogels are generally classified as physical hydrogels, while self‐crosslinking hydrogels are considered chemical hydrogels. Both types share advantages, including high biocompatibility and the ability to tune their formation and properties by adjusting molecular structures and preparation conditions. Regarding degradability, self‐assembling hydrogels, which rely on non‐covalent interactions, degrade gradually under physiological conditions triggered by environmental stimuli (e.g., pH, temperature, and ionic strength) or enzymatic action, offering excellent reversibility and dynamic responsiveness.^[^ ^141^ ^]^ Conversely, self‐crosslinking hydrogels, formed via covalent bonds, typically require harsher chemical or enzymatic conditions for degradation, resulting in slower degradation rates and greater suitability.^[^ ^142^ ^]^ Incorporating degradable ester bonds^[^ ^143^ ^]^ and disulfide bonds^[^ ^36^ ^]^ can effectively modulate the degradability of self‐crosslinking hydrogels, enabling controlled degradation for specific applications.

Previous reviews have categorized self‐assembling hydrogels based on their constituent materials, including peptide‐based, nucleic acid‐based, polysaccharide‐based, supramolecular‐based, and agglomerate‐derived hydrogels.^[^ ^144^ ^]^ Rather than detailing each category, we focus on its applications (**Table** **2**) and prospects.

**Table 2:** Key features and applications of self‐assembled hydrogels.

Types	Name	Component	Mechanism	Application	Refs.
Natural small molecule	Rhe@Ag Gel	Rhein; silver ions (Ag^+^)	π–π stacking; hydrogen bonding; Metal coordination	Infectious wound healing	[145]
	Rhein hydrogel	rhein	π‐π interactions; hydrogen bonds	Neural inflammation	[146]
Peptide‐Based	Nanofibers Gel	Fmoc–dipeptides ^a)^	Hydrogen bonding; p–p interactions	Three‐dimensional cell culture	[147]
	mPEG‐b‐poly(L‐alanine) (PEA) hydrogel	PEA copolymer	Hydrophobic and electrostatic interaction, Hydrogen bonding,	Tumor immunotherapy	[148]
Nucleic acid‐based	DNA–nSi injectable hydrogel	DNA;2D Silicate Nanodisks	Hydrogen bonding, Electrostatic interaction	Bone regeneration; Delayed/controlled release drug carrier	[149]
	HPMA/PNA hybrid hydrogel	N‐(2‐hydroxypropyl)methacrylamide (HPMA); Peptide nucleic acids (PNAs)	Hydrogen bonding	“drug‐free” therapeutics design	[150]
Polysaccharide‐Based	GC gels	Curdlan;Gelatin	Hydrophobic interaction; Hydrogen bonding	Oriented neural cell growth	[151]

^a)^ Fmoc: Fluorenylmethoxycarbonyl

**Figure 4:**
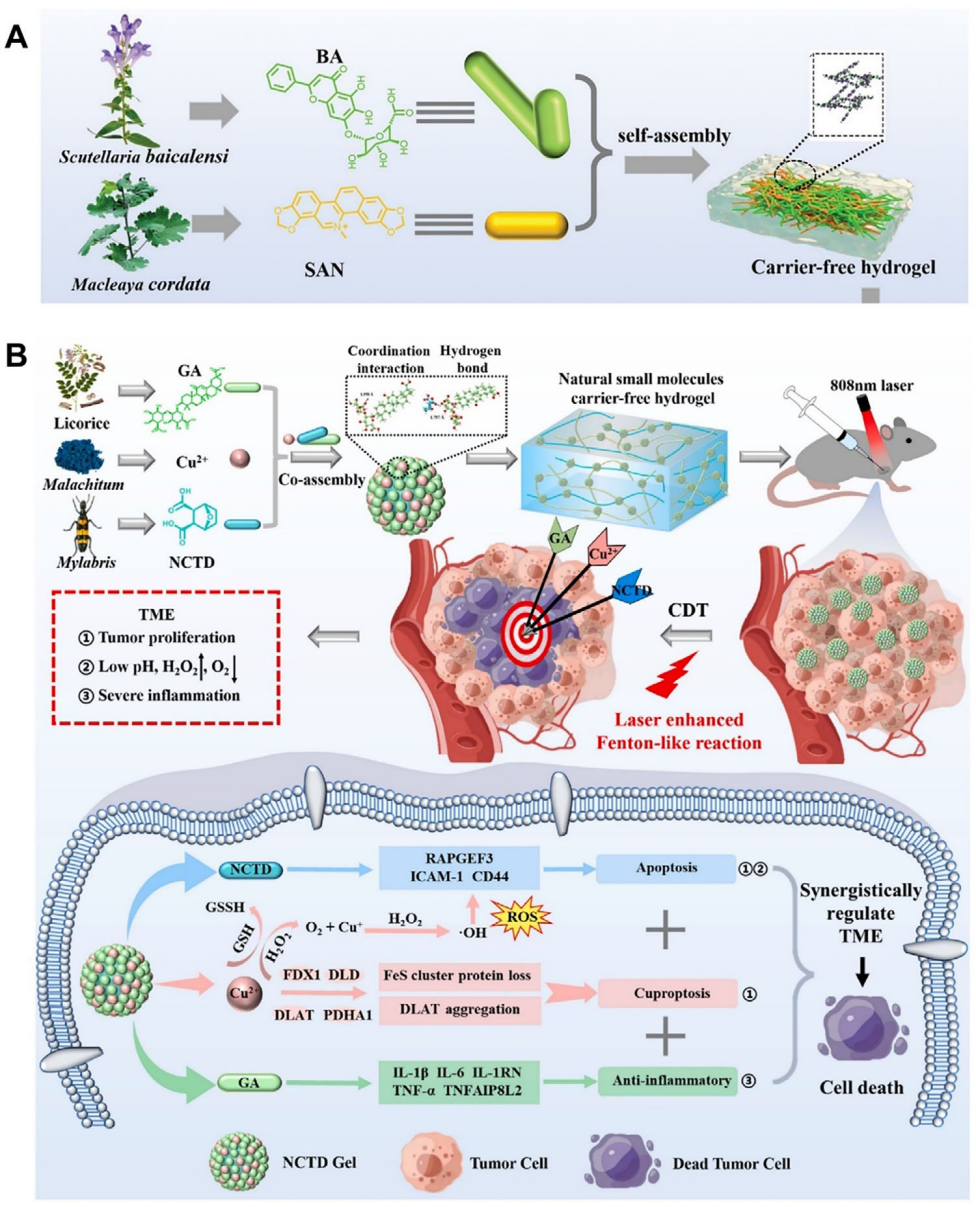
Innovative design strategies and applications of self‐assembled hydrogels. (A) Schematic illustration of the preparation of carrier‐free BA‐SAN hydrogel. Reproduced with permission.^[^ ^152^ ^]^ Copyright 2023, Wiley‐VCH. (B) Schematic illustration of NCTD Gel's self‐assembly mechanism synergistically regulates the tumor microenvironment via apoptosis, cuproptosis, and anti‐inflammation. Reproduced with permission.^154^ ^]^ Copyright 2023, Elsevier.

Recently, a carrier‐free hydrogel (BA‐Gel) was developed using phytochemicals baicalin (BA) and sanguinarine (SAN), leveraging their complementary anti‐inflammatory and antibacterial properties. The hydrogel was formed through self‐assembly driven by electrostatic attraction, π‐π stacking, and hydrogen bonding between BA and SAN.^[^ ^152^ ^]^ A dynamic network structure was formed through self‐assembly, providing physical support for wound healing. Additionally, the synergistic action of SAN and BA inhibited the production of bacterial virulence factors, thereby reducing inflammation triggered by these factors as antigens. In bacteria‐infected mouse models, BA‐Gel promoted wound healing by suppressing bacterial virulence, mitigating inflammation, and enhancing cell proliferation and tissue regeneration (**Figure** **4**A). This study introduced a clinically valuable antibacterial hydrogel, opening new avenues for designing carrier‐free hydrogels and small‐molecule phytochemicals for clinical applications.

Similarly, researchers created a super‐structured hydrogel (CHRgel) through the self‐assembly of hyaluronic acid (HA) and cordycepin (Cor) with an R‐peptide for healing surgical incisions post‐neoadjuvant radiotherapy.^[^ ^153^ ^]^ In vitro studies demonstrated that CHRgel enhanced the adhesion and proliferation of NIH‐3T3 cells and human umbilical vein endothelial cells (HUVECs), promoted angiogenesis, cleared radiation‐induced intracellular ROS, reduced DNA damage, and inhibited radiation‐induced senescence in HUVECs and NIH‐3T3 cells via the P21/P53 pathway. In vivo experiments confirmed that CHRgel significantly improved wound healing after radiotherapy. CHRgel is well‐suited for clinical translation and application with its straightforward composition and simple preparation process. Furthermore, self‐assembled hydrogels have shown promise in anti‐tumor applications.^[^ ^144^ ^]^ For instance, researchers developed a carrier‐free, injectable hydrogel (NCTD Gel) composed of Cu^2^⁺‐mediated self‐assembled glycyrrhizic acid (GA) and norcantharidin (NCTD), which is primarily formed through coordination and hydrogen bonding (Figure 4B).^[^ ^154^ ^]^ Under 808 nm laser irradiation, NCTD Gel generated ROS, depleted glutathione (GSH). It alleviated hypoxia in the tumor microenvironment (TME), thereby synergistically regulating the TME through apoptosis, cuproptosis, and anti‐inflammatory effects. The hydrogel's chemo‐dynamic therapy (CDT) exhibited high selectivity and biocompatibility, capitalizing on the TME's weak acidity and H_2_O_2_ overexpression. Notably, NCTD Gel's components are derived from clinical agents, and its preparation is simple, eco‐friendly, and cost‐effective, requiring no excipients. This study introduced a novel carrier‐free hydrogel strategy for synergistic anti‐tumor therapy, with strong potential for industrial production and clinical translation.

The mechanical properties of self‐assembled hydrogels, particularly their hardness and strength, remain limited. Innovative strategies have been developed that leverage multiple physical interactions or integrate dynamic covalent crosslinking (e.g., disulfide and imine bonds) with non‐covalent crosslinking to fabricate multi‐network hydrogels. This approach leverages the reversibility of non‐covalent bonds and the robustness of covalent bonds, allowing for the precise tuning mechanical properties of the hydrogel to achieve an optimal balance between toughness and rigidity. **Table** **3** summarizes the design and application of self‐crosslinking hydrogels. Precisely controlling and balancing the crosslinking degree and degradability of hydrogels is crucial for clinical translation.

**Table 3:** Key features and applications of self‐crosslinked hydrogels.

Name	Component	Mechanism	Condition	Application	Refs.
HA‐SH Gel	Thiolated hyaluronic acid derivative (HA–SH)	Free mercaptan groups oxidize to disulfide bonds	Exposed to air at 37 °C in 35 min (pH 7.4)	Anti‐tumor	[161]
HA‐DA Gel	Dopamine‐modified hyaluronic acid (HA‐DA)	autoxidation of Dopamine	10 µL of horseradish peroxidase (0.5 mg/mL) and 10 µL of H_2_O_2_ (10 mM)	Treatment of inflammatory bowel disease	[162]
Nanofibers Gel	Maleimidyl alginate; Pristine gelatin	Mild Michael type addition	Without any catalyst or external energy	Diabetic wound healing	[163]
ADAG Gel	Alginate dialdehyde borax solution (ADA); Gelatin	Schiff's base reaction	4‐min gelation in saline	Meniscal repair	[164]
HAC Gel	Sulfhydrylated hyaluronan (HA‐SH); type I collagen (Col I)	Sulfhydryl groups autoxidation	Aerobic condition; neutral pH, gel times at 150 s	Regulates chondrogenic differentiation of mesenchymal stem cells	[165]
DXR‐CBGel@aPD‐1	Sulfhydryl functionalized bovine serum albumin (BSA‐SH); maleimide (Mal) functionalized chitosan (CS‐Mal)	Michael addition (hydrosulphonyl, maleimide)	Mild	Inhibition of Postsurgery Malignant Glioma Recurrence	[166]

For the undefined self‐assembly or self‐crosslinking processes, their molecular structures and gelation mechanisms can be clarified through a combination of computational simulation and experimental verification.^[^ ^155^, ^156^ ^]^ For instance, full‐atom molecular dynamics simulation can reveal the atomic‐level pathways and interaction energies for the self‐assembly of short peptides into nanofibers,^[^ ^157^ ^]^ coarse‐grained modeling can simulate the formation kinetics of large‐scale polymer networks;^[^ ^158^ ^]^; quantum chemical calculations (such as density functional theory) can precisely predict the energy barriers and rates of key crosslinking reactions.^[^ ^159^ ^]^ The integration of these methods helps to understand the mechanism and ultimately improve the controllability of the degradation rate in self‐assembly/self‐crosslinking hydrogels.^[^ ^160^ ^]^

#### 2.1.2 Self‐Growing Hydrogel

It is similar to the phenomenon in nature, where organisms progressively grow and develop adaptive structures. Researchers are working to create self‐growing materials to achieve size growth, in situ post‐adjustment of properties, and unique adaptive structural growth. Currently reported growth materials are primarily categorized into four groups. The first is based on polymer network expansion,^[^ ^167^, ^168^, ^169^ ^]^ where monomers based on active groups in the main chain undergo homolysis to generate a free radical polymerization matrix. The latter is characterized by the presence of active groups (thiocarbonates, etc.) that can induce the formation of free radicals through photo‐stimulation, triggering polymerization and growth. The second is based on a polymer brush^[^ ^169^, ^170^, ^171^ ^]^ with active groups (thiocarbonate, α‐bromoester, hydroxyl, etc.) in the side chain of the network and using active free radical polymerization or ring‐opening polymerization to induce monomer‐to‐polymer conversion, generating a polymer brush structure to achieve growth. The third uses a covalent bond break to produce free radicals to induce monomer polymerization to achieve growth.^[^ ^172^, ^173^ ^]^ The fourth type hinges on the stimulus‐induced newly formed polymer network and the fusion with the original polymer network to achieve cyclic self‐growth.^[^ ^174^, ^175^ ^]^ Therefore, the self‐growing hydrogel exhibits reversible recombination and in situ growth characteristics, mimicking the growth strategy of organisms. Biological components such as cells, bacteria, and enzymes bring unique life characteristics to the self‐growing hydrogel.

At the macro level, controlling the growth rate and quality of self‐growing hydrogels is essential because rapid or sluggish growth rates can affect performance. The non‐uniformity in the growth process may also lead to structural defects and performance degradation of hydrogels. Although some self‐growing hydrogels have achieved post‐adjustment of gel properties and size to a certain extent, self‐growing hydrogels still have certain limitations in property regulation and continuous growth due to their cross‐linked structures, which lead to swelling limitations and dynamic cross‐linked structures requiring additional stimulation to achieve multiple swelling growth. To address these challenges, researchers proposed an interfacial free radical polymerization growth model inspired by keratin autogrowth,^[^ ^176^ ^]^ which is characterized by the continuous free radical polymerization of liquid metal (*i.e*., eutectic indium gallium, EGaIn) and methyl acrylamide (AAm) monomer aqueous solution at the interface. The newly formed hydrogel shows the characteristics of continuous regeneration. In vitro studies have demonstrated that polyacrylamide (PAAm) hydrogels can self‐grow up to approximately 80 mm on glass tubes without additional high‐energy input. Staining the AAm monomer precursors with traceable indicators revealed that the hydrogels can also spontaneously grow along pre‐specified microscale channels, consuming all AAm precursors within ≈15 h. This self‐growth behavior of hydrogels presents significant promise in soft‐walled climbing robot applications, evidencing notable advantages in unclogging small, curved pipelines, especially for microchannels where large‐scale machinery cannot be utilized. Furthermore, at the microscopic dynamics level, researchers proposed a chemo‐mechanical model of mechanically reactive self‐growing hydrogels by developing and integrating the theories of chain break mechanical generation,^[^ ^177^ ^]^ polymerization chemical kinetics,^[^ ^172^ ^]^ and new network formation.^[^ ^178^, ^179^ ^]^ The model has been applied to theoretically investigate the concentration of mechanical radicals generated during the stretching of hydrogels. The polymerization kinetics of monomers and cross‐linking agents, as well as the enhanced mechanical behavior of self‐growing hydrogels resulting from the formation of new networks, are consistent with the experimental measurements of Matsuda et al.^[^ ^172^ ^]^ Both can explain the mechanical behavior of self‐growing hydrogels from the microscopic level.

Self‐assembly, self‐crosslinking, and self‐growing hydrogels spontaneously form according to their material properties and internal mechanisms. Its design and fabrication represent a complex and intricate process, requiring careful consideration of factors such as gelators, solvents, molecular structure modifications, mixing methods, gelation mechanisms, as well as pH, temperature, ionic strength, and ion types in the sol state, to achieve optimal performance and stability. Currently, the field still faces several challenges, such as insufficient control over assembly pathways, low predictability of crosslinking processes, and structural instability or performance failure caused by environmental fluctuations in practical applications. To address these issues, future research will focus on employing multiscale modeling and machine learning methods to precisely regulate assembly and crosslinking processes, enhance material stability and self‐regulatory capabilities through dynamic covalent chemistry and adaptive networks, and leverage orthogonal crosslinking strategies and bio‐inspired designs (e.g., multi‐network topologies) to balance mechanical strength and self‐healing properties.

Building on this foundation, scientists have gained a deeper understanding of the interactions between the intrinsic properties of materials and their external environments, laying the groundwork for the development of more advanced adaptive systems. The “external” adaptive properties of hydrogels are explored to reveal the unique advantages of adaptive, self‐regulating, and self‐adhering hydrogels in response to external stimuli and multi‐functional integration, providing more comprehensive theoretical support and practical guidance for future smart material design.

### 2.2 Adaptive Hydrogels/Self‐Regulating Hydrogels

Dynamic responsiveness and reversibility are the defining characteristics of adaptive or self‐regulating hydrogels. By engineering network architectures with dynamic bonds, stimuli‐responsive moieties, or intelligent molecules, adaptive or self‐regulating hydrogels can respond to external stimuli (e.g., temperature, pH, light, mechanical force, and molecular concentration) or internal changes (drug release demands, cell growth, etc.).^[^ ^180^, ^181^, ^182^ ^]^ They autonomously modulate their physicochemical properties through integrated feedback mechanisms, including structural topology, swelling behavior, mechanical compliance, or drug release kinetics, to achieve macroscopic self‐adaptation, self‐regulation, or self‐adhesion in response to evolving environmental conditions or application‐specific requirements. This potential to mimic the complex and adaptive behavior of natural tissues has attracted keen interest in biomedical engineering.^[^ ^183^ ^]^

#### 2.2.1 Adaptive/Self‐Adjusting Hydrogels

**Figure 5:**
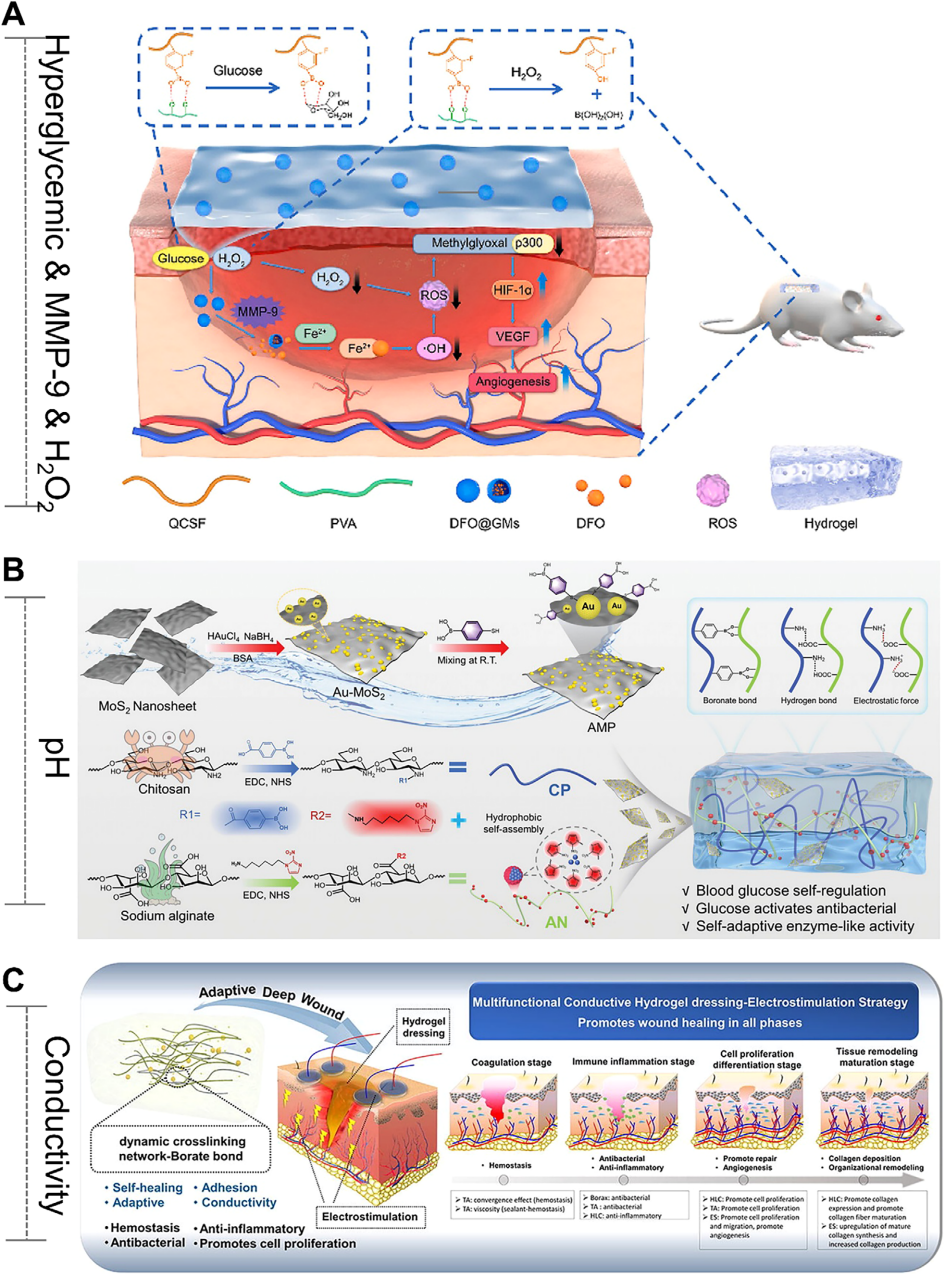
Smart Hydrogels with Microenvironment‐Adaptive Properties: Design and Applications. (A) Mechanistic illustration of the self‐adaptive DFO@G‐QCSFP hydrogel for diabetic wound therapy. Reproduced with permission.^[^ ^184^ ^]^ Copyright 2023, Elsevier. (B) Schematic overview of the programmable CPAN─AMP hydrogel (CP/AN/AMP) activated by glucose for infected diabetic wound repair. Reproduced with permission.^[^ ^190^ ^]^ Copyright 2025, Wiley‐VCH. (C) The role of combination therapy in facilitating complete wound healing. Reproduced with permission.^[^ ^191^ ^]^ Copyright 2021, Elsevier.

Unlike the self‐activated hydrogels (Section 2.7.1) that focus on triggering and executing one‐way programmed responses based on “causes”, adaptive/self‐adjusting hydrogels place greater emphasis on closed‐loop regulation of “effects”. The core functionality relies on continuously adapting its state and function through dynamic, reversible interactions in response to environmental changes, ultimately establishing a self‐correcting “stimulus–response–feedback–adaptation” cycle. In particular, they can dynamically sense key signals in the pathological microenvironment (such as high blood sugar, reactive oxygen levels, pH fluctuations, and changes in enzyme activity), and thereby achieve intelligent drug release and precise functional regulation, providing innovative strategies for the precise treatment of chronic wounds in diabetes and other complex diseases. For instance, a glucose/hydrogen peroxide/matrix metalloproteinase (MMP) responsive hydrogel was developed explicitly for diabetic wounds.^[^ ^184^ ^]^ This hydrogel was achieved by modifying the dynamic borate ester bond between chitosan and polyvinyl alcohol. The aim was to combat oxidative stress and vascular dysplasia by releasing deferoxamine (DFO) in two stages. In the early stage of wound healing, the borate ester bond in the hydrogel interacted with high glucose and hydrogen peroxide, thereby alleviating oxidative stress and releasing DFO@G. Subsequently, the sustained release of DFO is achieved through the response to overexpressed matrix metalloproteinases. In diabetic mice, the hydrogel promoted angiogenesis (via HIF‐1α and growth factors), collagen deposition, and accelerated wound healing, demonstrating its potential for treating chronic wounds (**Figure** **5**A). These hydrogels are often loaded with therapeutic agents, such as gelatin microspheres containing celecoxib (GMs@Cel) and insulin (INS),^[^ ^185^ ^]^ angiogenesis‐promoting deferoxamine (DFO@G),^[^ ^184^ ^]^ or composite materials like metformin, l‐arginine, and l (+)‐ascorbic acid liposomes,^[^ ^186^ ^]^ which release encapsulated drugs in a microenvironment‐controlled manner. For example, Zhou et al. developed glucose/MMP‐9 dual‐response temperature‐sensitive hydrogel loaded with celecoxib (GMs@Cel) and insulin (INS). The gel exhibited fluidity at 37 °C, allowing it to fill deep wounds, and solidified at 25 °C to protect the wound from mechanical stress, enabling on‐demand drug release and significantly accelerating healing.^[^ ^185^ ^]^ Xie et al. designed an antioxidant carboxymethyl cellulose hydrogel with bioinspired wet adhesion, adapting to dynamic wound deformation.^[^ ^187^ ^]^ The authors constructed a pH‐responsive hyaluronic acid hydrogel to actively adjust the alkaline microenvironment of diabetic wounds to a healing‐friendly acidic state by modifying hyaluronic acid and poly (6‐aminocaproic acid).^[^ ^188^ ^]^

Beyond multi‐signal coordination and ROS scavenging, dynamic feedback‐regulated therapeutic systems demonstrate remarkable potential. A programmable adaptive hydrogel composed of phenylborate‐modified chitosan (CP), hydrophobic and hypoxia‐sensitive 2‐nitroimidazole‐modified alginate (AN), and phenylborate‐functionalized Au MoS_2_ complex nanase (AMP) was developed. AMP nanase can adapt to the wound microenvironment's pH, self‐switching of various enzyme activities, and, more importantly, insulin can be released in a dynamic feedback manner, which enables the dual adaptation of blood glucose regulation and wound healing promotion (Figure 5B). Alternatively, Zheng et al. developed an injectable Ca^2^⁺/pH dual‐responsive fibrin/mesoporous bioactive glass/alginate (SMS) hydrogel. By sensing calcium ion concentrations and inflammatory pH in bone defect microenvironments, the hydrogel adaptively adjusts its mechanical strength and degradation rate, enabling seamless cavity filling.^[^ ^33^ ^]^ Liu et al. designed a microenvironment‐responsive hydrogel loaded with puerarin (PUE) to scavenge ROS and reverse hypoxia in infarcted areas. Combined with its ability to maintain myocardial stem cell “stemness,” this hydrogel promotes functional recovery in the infarction area.^[^ ^189^ ^]^ While electrical conductivity (e.g., polyaniline and graphene) is not an inherently “adaptive” property, its integration into hydrogels can mimic the natural tissue microenvironment to enhance cell migration and signal transduction, ultimately promoting electrophysiological repair.

Self‐adaptive hydrogels, operating through a “sense‐feedback‐regulate” closed‐loop mechanism, exhibit transformative potential in diabetic wound healing, bone regeneration, and myocardial repair. In the future, through multi‐response collaboration, bionic design, and clinical adaptation, they are expected to become an innovative carrier for personalized treatment of complex diseases.

#### 2.2.2 Adaptive Conductive Hydrogel

Most current hydrogel wound dressings can only be applied topically to deep wounds, preventing endogenous and external current conduction, which is not conducive to wound healing; hence, the development of adaptive conductive hydrogels.^[^ ^183^, ^192^, ^193^, ^194^ ^]^

For instance, Lei *et al.* successfully developed an adaptive conductive hydrogel for wound healing applications.^[^ ^195^ ^]^ This hydrogel incorporated tannic acid (TA) and human‐like collagen (HLC) into a dynamic cross‐linked network of polyvinyl alcohol (PVA) and borax hydrogel. The conductivity of the hydrogel is derived from borax, which serves as both a crosslinking agent and an ionic conductor. The migration of borax ions in an aqueous environment enables the hydrogel to achieve a conductivity range of 1 × 10^15^ to 0.26 S/m, closely matching human skin conductivity. This ionic conductivity, combined with the dynamic hydrogen and borate bonds, imparts the hydrogel with adaptability, self‐healing capabilities, and electrical responsiveness. Furthermore, the incorporation of HLC and TA enhances the hydrogel's multifunctionality, providing hemostatic, antibacterial, anti‐inflammatory, and antioxidant properties. These components also promote fibroblast proliferation and collagen expression, critical for effective wound repair. (Figure 5C). In a rat full‐thickness skin defect model, the hydrogel demonstrated excellent conformability to deep wound cavities, facilitating the conduction of endogenous and exogenous electrical currents. This enhanced intercellular signaling, cell migration, and angiogenesis. By day 10, the hydrogel achieved complete wound closure with significant reconstruction of subcutaneous tissue, including blood vessels and pores.

Adaptive/self‐regulating hydrogels have broad application prospects and excellent development potential. In particular, the combination of adaptive conductive hydrogels with electrical stimulation is a promising strategy for treating deep wounds. However, they encounter problems of stability decline and performance attenuation during long‐term use. Dynamic covalent chemistry, stimulus‐response network optimization, and biocompatible material design, combined with the integration of synthetic biology or nanotechnology, are expected to facilitate the development of multifunctional hydrogel systems.

### 2.3 Self‐Healing Hydrogel

A self‐healing hydrogel is a network of low‐molecular‐weight molecules or macromolecules cross‐linked reversibly via dynamic covalent reactions (chemical cross‐linking) or non‐covalent bonds (physical cross‐linking) with water as a continuous phase.^[^ ^196^ ^]^ Amongst reversible covalent bonds used in the construction of self‐healing hydrogels are boronate ester,^[^ ^197^, ^198^ ^]^ disulfide,^[^ ^199^ ^]^ metal‐ligand coordination,^[^ ^200^ ^]^ Schiff base,^[^ ^201^ ^]^ and bonds generated via cycloaddition reaction.^[^ ^202^ ^]^ The reversible nature of cross‐links causes a broken network to be regenerated by rebuilding the cross‐links. Among non‐covalent interactions that form self‐healing hydrogels, hydrogen bonding,^[^ ^203^ ^]^ ionic,^[^ ^204^ ^]^ hydrophobic,^[^ ^197^ ^]^ and host‐guest interactions^[^ ^205^ ^]^ can be distinguished. The network can be constructed of two kinds of interactions, generating a double network. For example, a hydrogel network can be created from both covalent and non‐covalent bonds,^[^ ^206^, ^207^ ^]^ two kinds of covalent cross‐links,^[^ ^208^ ^]^ or two non‐covalent interactions.^[^ ^209^ ^]^ The regeneration of cross‐links is possible due to the reversible nature of cross‐links and the molecular mobility of the network chains.^[^ ^210^ ^]^

#### 2.3.1 Injectable and Self‐Healing Hydrogels

**Figure 6:**
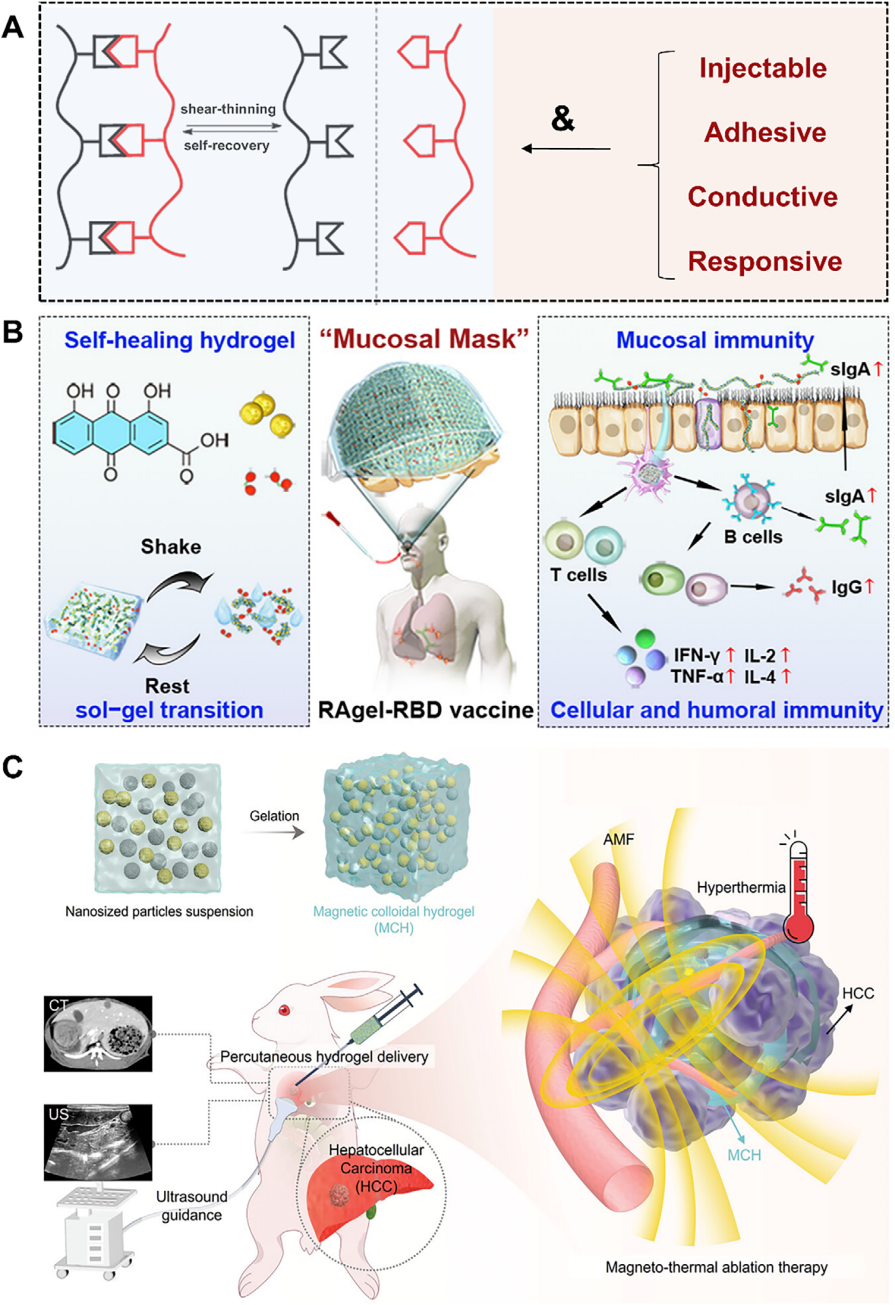
Advanced injectable self‐healing hydrogels and their applications. (A) Shear‐thinning and self‐healing hydrogels for injectable delivery and multifunctional customization. (B) Schematic of RAgel‐RBD for intranasal immunization. Al^3^⁺‐induced reversible RAgel enables antigen loading and sol–gel transition. Post‐administration, it acts as a “mucosal mask” to protect antigens and prolong nasal/lung residence, enhancing APC uptake. This stimulates cytokine release, immune cell recruitment, and mucosal IgA/IgG responses against SARS‐CoV‐2. Reproduced with permission.^[^ ^212^ ^]^ Copyright 2024, ACS Publications. (C) The application method of injectable magnetic colloidal hydrogel (MCH) for ultrasonic‐guided magnetic hyperthermia ablation of hepatocellular carcinoma (HCC). Ultrasound guidance is used for minimally invasive implantation of MCH, followed by magnetic hyperthermia ablation of HCC under the action of an alternating magnetic field (AMF). Reproduced with permission.^[^ ^214^ ^]^ Copyright 2024, Wiley‐VCH.

The hydrogels constructed based on reversible crosslinking structures often exhibit self‐healing ability and shear‐thinning properties due to the reversible behavior of the bond breakage and reformation. The reversibility of this crosslinking structure ensures the shear‐thinning property. During the injection process, as the shear rate increases, the crosslinking structure is disrupted, and the viscosity of the hydrogel decreases, causing the hydrogel to transform from a gel state to a liquid state.^[^ ^200^, ^203^, ^206^, ^211^ ^]^ After the injection is completed, the original viscosity will recover. Due to the recombination of the broken crosslinking structures, the gel state of the hydrogel will rapidly recover (**Figure** **6**A).^[^ ^206^ ^]^

The injectable character of hydrogels is crucial for their administration to hard‐to‐reach sites, typical of many biomedical applications. The hydrogel could be effectively injected into the injury site in a minimally invasive way to fill the lesion cavity or ensure the controlled release of encapsulated therapeutics. Notably, injectable hydrogels combine adhesion, conductivity, and responsiveness to stimuli, thereby expanding their biomedical applications. Since the mechanical properties of hydrogels can be tailored to resemble a range of natural nerve tissues and modulate stem cell proliferation and differentiation, they are promising biomaterials for nervous system therapy. The hydrogel, constructed from pyrrole‐grafted hyaluronic acid (Py‐grafted HA) and ferric ions, was injected directly into the rat sciatic nerve crush injury sites for nerve regeneration in vivo and was found to promote functional recovery and remyelination.^[^ ^169^ ^]^ Moreover, injectable hydrogels constructed of borax‐functionalized oxidized chondroitin sulphate (BOC), BOC‐doped polypyrrole (BOCP), and gelatin (Gel) via covalent Schiff‐base and borate‐diol ester bonds and noncovalent electrostatic interactions can effectively fill the lesion cavity and conform to the shape of the defect in the case of traumatically injured spinal cord, seamlessly integrating with the host tissue.^[^ ^178^ ^]^

Furthermore, by utilizing two clinically verified, practical components, namely the dimer antigen of the receptor binding domain (RBD) and the SARS‐CoV‐2 piercing protein (RBD‐dimer), and through the coordination of the phenolic hydroxyl group and aluminum ions of emodin, an injectable self‐healing hydrogel mucosal vaccine (RAgel) was prepared. It forms an adhesive, protective film on the surface of the nasal mucosa, known as a “mucosal mask”. This “mucosal mask” stabilizes the antigen and prolongs its residence in the nasal cavity and lungs. It can also stimulate the recruitment and maturation of immune cells, promote antigen internalization and cross‐presentation, enhance the immune response of the mucosa, and prevent respiratory virus infections, especially COVID‐19 caused by severe acute respiratory syndrome coronavirus 2 (SARS‐CoV‐2). Compared with traditional methods, RAgel does not require chemical modification, is easy to prepare, and is more easily converted into therapeutic drugs. The design and application of self‐healing hydrogel vaccines offer a new direction for future vaccine research and development, particularly for vaccines that require administration through the mucosal route. Additionally, researchers have developed a biomaterial based on the myocardial ECM. After injection, it can gel and prevent myocardial infarction and post‐infarction scar formation by enhancing contractile function and contractile force, playing a significant role in cardiovascular regeneration (Figure 6B).^[^ ^212^ ^]^

In recent years, the trigger gelation strategies for injectable hydrogels can be mainly classified into two categories. The first one is physical gelation, such as the temperature‐sensitive polymer PNIPAM, which induces phase transition through dynamic temperature regulation of physical interactions (such as hydrogen bonds and hydrophobic interactions), achieving *in‐situ* gelation and self‐repair after injection, with strong self‐healing ability and no need for external triggering. This shows unique advantages in injectable self‐healing hydrogels and enables multiple injury‐healing cycles.^[^ ^213^ ^]^ The second type is chemical gelation hydrogels based on biopolymers (such as alginate, hyaluronic acid, gelatin) and their derivatives (such as methacrylate), which usually require chemical crosslinking methods such as photopolymerization to trigger gelation, but these systems face significant challenges in clinical translation: the dependence of the phototrigging system on ultraviolet light limits its application range, the cytotoxicity of free radical initiators and the biological safety issues of long‐term ultraviolet exposure need to be addressed, especially in deep tissue (such as liver cancer) treatment, where the accessibility of short‐wave light is inherently limited. This prompts researchers to explore infrared or near‐infrared trigger systems to expand the application of phototrigged injectable hydrogels in deep tissues. In contrast, the researchers reported the magnetic colloidal hydrogel (MCH) composed of Fe_3_O_4_ and gelatin through electrostatic self‐assembly, which exhibits unique advantages: this system achieves rapid self‐repair and percutaneous injectability through reversible electrostatic crosslinking, avoiding the defect of traditional covalent crosslinked hydrogels requiring surgical implantation; it also has excellent magnetic thermal response properties and biocompatibility, and has been confirmed to have significant efficacy in H22 tumor mouse models and large animal deep liver cancer (HCC) models. The MCH hydrogel innovatively combines the deep penetration advantage of magnetic hyperthermia (MHT) with minimally invasive puncture technology, overcoming the limitations of phototrigged gels in deep tumor treatment, not only achieving non‐invasive precise treatment for early unresectable liver cancer, but also effectively inhibiting tumor growth and reducing the risk of recurrence through remote local heating, while avoiding the damage to normal tissues caused by high‐field strength long‐term ablation, providing a new minimally invasive strategy for clinical ablation treatment of liver cancer (Figure 6C).^[^ ^214^ ^]^

#### 2.3.2 Self‐Healing and Injectable Hydrogels with Conductive Properties

Conductive hydrogels are emerging as powerful platforms for biomedical applications, including flexible sensors, wearable devices, and wound dressings. The conductive hydrogels facilitate the detection of multiple parameters, such as body motions^[^ ^171^ ^]^ or microbial infections.^[^ ^206^ ^]^ The interest in conductive hydrogels results from incorporating electroactive materials into the skin that transmit bioelectrical signals, promoting skin cell proliferation, migration, and adhesion, thereby accelerating wound healing, *i.e*., tissue regeneration,^[^ ^215^ ^]^ particularly in chronic wounds.^[^ ^216^ ^]^ Thus, conductive hydrogels are promising systems in dressings for chronic diabetic foot ulcers.^[^ ^217^ ^]^ Moreover, hydrogels with high electroactivity can enhance neuronal proliferation and axonal extension. Thus, they can be a key material in the therapy of nerve tissue regeneration, for instance, traumatic spinal cord injury.^[^ ^218^ ^]^

Conductive hydrogels can be constructed of polypyrrole,^[^ ^204^ ^]^ polyaniline,^[^ ^93^ ^]^ or conductive particles such as multiwalled carbon nanotubes,^[^ ^219^ ^]^ hydroxylated graphene,^[^ ^206^ ^]^ and MXene@polydopamine nanosheets.^[^ ^220^ ^]^ Shen et al. constructed the conductive hydrogels using two parallel conducting compounds, polypyrrole‐grafted gelatin, and ferric ions.^[^ ^204^ ^]^ The conductivity of PPy‐GelMA‐Fe hydrogel was higher (≈16 mS/cm) than that of single‐based systems, i.e., GelMA‐Fe and PPy‐GelMA hydrogels with ≈10 mS/cm. Li et al. built the conductive hydrogel by introducing reduced graphene oxide coated with polydopamine (rGO‐PDA) into the network with quaternized chitosan.^[^ ^221^ ^]^ The conductivity of prepared hydrogels gradually enhanced to 5.0, 5.1, 5.3, and 5.6 mS/cm upon the gradual increase of rGO‐PDA content to 1, 2, 3, and 4 mg/mL, respectively. The hydrogel constructed of the hydroxylated graphene with aminophenylboronic acid grafted sodium alginate (Alg‐PBA) assured the detection of the motion of stretchable body parts (elbows, knees, fingers, wrists, etc.) and the detection of bacteria in the wound which presence caused the acidification and the reduction of the cross‐linking density of the hydrogel demonstrated by the increase of resistance,^[^ ^206^ ^]^ facilitating treatment without the concern of increasing inflammation. This was of great importance in the case of healing chronic wounds, as it prevented re‐tearing and accelerated wound healing. Thanks to advantageous characteristics of multiwalled carbon nanotubes, such as electrical conductivity, biocompatibility, high antimicrobial activity, high drug loading capacity, and high mechanical strength, the multiwalled CNT‐loaded hydrogel obtained via cross‐linking of four‐armed SH‐PEG with Ag^+^ via Ag–S coordinates succeeded as carriers of metformin and adipose‐derived stem cells (ADSCs) in the therapy of chronic diabetic wounds.^[^ ^219^ ^]^ **Table** **4** presents exemplary hydrogel designs that ensure self‐healing, injectable properties, and adhesive or conductive properties.

**Table 4:** Advances in injectable, self‐healing hydrogels: Mechanisms and their role in tissue regeneration and therapeutic delivery systems.

Name	Component	Mechanism	Properties	Application/stages of biological investigation	Refs.
HASPy Gel	Hyaluronic acid modified with cysteamine and pyrrole‐1‐propionic acid/FeCl_3_	Chain‐connecting Fe bonds, hydrogen bonds, disulfide bond	Injectable, self‐healing conductive	Promotion of peripheral nerve regeneration; In vivo study	[199]
QCS‐PA@Fe Gel	Dual‐dynamic‐bond cross‐linking among Fe, PA containing catechol, aldehyde groups, and QCS	Catechol‐Fe and Schiff base bonds	Injectable, self‐healing adhesive	On‐Demand Removability for Post‐Wound‐Closure and Infected Wound Healing fimbriae; In vivo study	[200]
LGO Gel	L‐(propylthio)acetic‐3‐butylimidazole‐modified poly(L‐lysine) (PLL‐PBIM)/adipate dihydrazide‐modified poly(L‐glutamic acid) (PLG‐ADH) /oxidized dextran (ODex)	Schiff base bonds	Injectable, sprayable, self‐healing adhesive	Antibacterial properties of *Staphylococcus aureus* and *Escherichia coli* wound dressing; In vivo study	[201]
Alg‐PBA/PVA/GOH Gel	Aminophenylboronic acid grafted sodium alginate/poly(vinyl alcohol)/hydroxylated graphene (GOH)	Borate ester, Supramolecular interactions	Injectable, self‐healing, conductive, motion monitoring, antibacterial	Detection of bacterial infection and killing bacteria; wound dressing for joints In vitro and in vivo study	[206]
PL‐CD @ ODA Gel	Poly(L‐lysine) carbon dot (P‐CD)/oxidized dextran (ODA)	Schiff base bonds	Injectabl,e, self‐healing antibacterial	Antibacterial properties against *Staphylococcus aureus;* In vitro study	[222]
ACCP Gel	Aldehyde‐based hyaluronic acid (ALHA)/polyaniline‐modified‐carboxymethyl chitosan (PANI‐CMCS)	Schiff base bonds	Injectable self‐healing conductive	Promote peripheral nerve regeneration and motor functional recovery; In vivo study	[223]
aCS‐aAlg‐DNH Gel	Aldehyde‐alginate (aAlg) /acrylic acid‐chitosan (aCS) /adipic acid dihydrazide (DNH) and FeCl_2_	Double network: Schiff base and ionic interactions	Self‐healing injectable degradable	Doxorubicin/Ciprofloxacin/cells delivery systems; In vitro study	[224]
CSMA/SC Gel	Chondroitin sulfate multiple aldehyde (CSMA)/N‐succinyl‐chitosan (SC)	Schiff base	Injectable, self‐healing	Cell encapsulation, In vitro study	[225]
QCS/TA Gel	Quaternary ammonium chitosan (QCS)/Tannic acid (TA)	Ionic bonds hydrogen bonding	Injectable self‐healing adhesive	Reactive oxygen species scavenging activity, broad‐spectrum antibacterial, and rapid hemostatic capabilities; In vivo study	[226]
CMC‐Eu‐EDTA Gel	Carboxymethyl cellulose (CMC) with pre‐coordinated europium‐ethylenediaminetetraacetic acid (Eu‐EDTA)	Metal‐carboxyl coordination	Self‐healing injectable pH monitoring	Monitor and treat diabetic wounds, In vivo study	[97]
P(NIPAM_290_‐*stat*‐AH_27_)/pectin‐CHO Gel	Aldehyde‐modified pectin(pectin‐CHO)/acylhydrazide‐functionalized poly(N‐isopropylacrylamide‐stat‐acylhydrazide) (P(NIPAM‐stat‐AH))	Acylhydrazone bond	Injectable biodegradable self‐healing	Controlled drug carrier to CT26 tumour, In vitro and vivo study	[227]
Poly(NIPAM‐co‐β‐CD)/CNT/PPY Gel	Poly(β‐cyclodextrin acrylate‐NIPAM), CNT, polypyrrole nanoparticles	Host–Guest Interactions	Self‐healing injectable conductive NIR‐responsive	Poly(NIPAM‐co‐β‐CD)/CNT/PPY hydrogel displayed conductivity up to 34.93 S/m; In vitro study	[205]

### 2.4 Self‐Powered Hydrogels

Self‐powered hydrogels belong to another category of programmable hydrogels featuring advanced polymer networks with intrinsic coupling between their molecular structure and electric energy, enabling energy harvesting, storage, and sensing capabilities.^[^ ^228^, ^229^, ^230^, ^231^, ^232^ ^]^ These hydrogels can generate electrical energy in response to external mechanical or environmental stimuli through triboelectric,^[^ ^229^, ^232^, ^233^, ^234^, ^235^, ^236^, ^237^, ^238^ ^]^ piezoelectric,^[^ ^239^, ^240^, ^241^ ^]^ or ion‐conductive coupling mechanisms.^[^ ^242^, ^243^ ^]^ This unique functionality highlights their translational potential for self‐powered sensors, capacitors, and actuators across various applications, including wearable electronics, environmental monitoring, and biomedical devices. The inherent mechanical compliance of self‐powered hydrogels is crucial for successfully integrating them into these novel applications. In biomedicine, these properties ensure minimal invasiveness and seamless interaction with delicate biological tissues. Flexibility is essential for comfortable and unobtrusive device operation in wearable electronics. Biocompatibility is another cornerstone for the safe and effective use of self‐powered hydrogels within living systems, ensuring that the materials do not elicit adverse biological responses (i.e., improving acceptability) and allowing for long‐term, reliable operation within the human body. Self‐powered hydrogels exhibit remarkable responsiveness to external stimuli. They dynamically adjust their electrical output in response to temperature, pH, or light exposure, to name a few stimuli, and adapt to environmental conditions, and perform complex tasks. Finally, the ability of some self‐powered hydrogels to self‐heal after damage significantly enhances their durability and longevity. The intrinsic repair mechanisms mitigate the impact of wear and tear, extending these materials' operational lifespan (number of cycles) and reducing the need for frequent replacements, particularly in demanding applications. For example, a multifunctional sensing platform demonstrating integrated tactile‐visual perception was developed using an asymmetric bilayer ionic hydrogel configuration. It achieves self‐powered multimodal sensing through the piezoionic effect and ionic thermodiffusion effect (with minimum pressure/light intensity detection limits of 3.6 kPa and 35.7 mW cm^−2^, respectively), while simulating synaptic plasticity (including short‐term and long‐term potentiation) via ion relaxation effects caused by the mobility difference between anions and cations. The device enables information writing and reading without an external power supply, and its skin‐retina‐like signaling pathway empowers robotic arms with intelligent grasping and danger‐avoidance capabilities, establishing a new paradigm for developing neuromorphic sensory integration devices in humanoid robotics.^[^ ^244^ ^]^

#### 2.4.1 Smart Hydrogel Triboelectric Nanogenerator Modes

Hydrogel triboelectric nanogenerators (*h*TENG) are distributed mechanoelectrical devices that convert mechanical energy into electrical energy based on the coupling effect of contact electrification (electron transport) and electrostatic induction (charge redistribution), which stems from mechanical contact and friction mechanisms (previously viewed as lost energy).^[^ ^234^, ^245^, ^246^ ^]^ Contact electrification is based on direct contact and separation between different hydrogel materials, resulting in electron transfer and changing the net polarization in both materials.^[^ ^245^, ^247^, ^248^ ^]^ On the other hand, electrostatic induction involves redistributing electric charges within a material without direct contact due to the influence of a nearby charged object.^[^ ^245^, ^248^, ^249^ ^]^

To address the treatment challenge of peroneal nerve injury (PNI), Zhou *et al.* developed a self‐powered neural stimulation system based on the triboelectric effect. This system converts the biological mechanical energy from respiratory movements into electrical signals through an alternating friction structure of polydimethylsiloxane (PDMS) and polyamide 6 (PA6) films, driving the nerve cuff electrodes to generate electrical stimulation that matches the natural nerve pulses. In a rat model, this system significantly promoted nerve regeneration and functional recovery, with no tissue side effects. This design approach of energy harvesting and dynamic adaptation to the biological environment provides valuable insights for the application of intelligent programmable hydrogels in nerve repair.^[^ ^250^ ^]^ Pei et al. highlighted the potential of melanin‐like nanoparticles in enhancing the mechanical, triboelectric, and environmental performance of biodegradable *h*TENGs for durable and eco‐friendly wearable applications, accurately detecting a range of human movements, including walking, jogging, and jumping.^[^ ^251^ ^]^ The addition of 0.4 *wt*.% melanin‐like nanoparticles resulted in a tenfold increase in elongation at break, a 13.2× increase in tensile toughness, an 11% rise in tensile strength, and a nearly twofold increase in charge density (414.88 µC/m^2^).^[^ ^251^ ^]^ Yang et al. also reported the development of liquid metal‐doped hTENGs with high‐performance, stability, and biocompatibility, TENGs for wearable and self‐powered sensing applications, achieving *V*_OC_ ≈ 223.32 V, *I*_SC_ ≈ 9.78 µA, *Q*_SC_ ≈ 82.33 nC, and power density of 4.30 W/m^2^.^[^ ^252^ ^]^ Xie et al. prepared a polyvinyl alcohol (PVA) /polyacrylamide (PAAM)/ polydiethylthiophene (PEDOT) three‐network hydrogel (PMPZ) through Zn^2^⁺ adsorption and semi‐interpenetrating network design. This hydrogel exhibits excellent mechanical properties (tensile strength of 83.79 kPa, compressive strength of 96 MPa) and high sensitivity (strain coefficient of 48.6). Based on this, the PMPZ‐TENG was developed, which can achieve an open‐circuit voltage of 326.89 V and a power density of 368.3 µW/cm^2^ under a 100 MΩ load. It also possesses flexibility, integrability, and operational stability, and has significant potential for human health monitoring applications, providing a promising material strategy for the development of high‐performance flexible electronic devices.^[^ ^253^ ^]^ Xu et al. investigated self‐powered *h*TENG wound dressing and tracking different physiological motions that can generate up to 50 V and an output current of 1.2 µA through gas‐solid contact‐separation, where the triboelectric stimulation enhances keratinocyte proliferation by 35%, migration by 40%, and adhesion by 30% compared to control groups.^[^ ^254^ ^]^ The sc‐*h*TENG device exhibited a 25% faster re‐epithelialization rate and a 20% increase in collagen deposition compared to traditional methods during in vivo experimentation. This hydrogel framework maintained stable performance over 100k deformation cycles and retained 95% of its initial mechanical properties after 30 days of continuous use.

The single electrode mode *h*TENG (SE‐*h*TENG) unlocks the translation potential in motion trackers, health electronics, robotics, human‐machine interfaces, emergency devices, E‐skins, self‐powered sensors (pressure, heat, etc.), material identifiers, health devices, and biomechanical energy harvesters.^[^ ^46^, ^255^, ^256^, ^257^, ^258^, ^259^, ^260^, ^261^ ^]^ SE‐*h*TENG benefits include simplified device configurations (single electrode construction and eliminating cumbersome wiring) and efficient self‐powering.^[^ ^255^ ^]^ Cong et al. introduced a method for mass‐producing *h*TENGs using cost‐effective and scalable dispenser printing without requiring complex equipment or procedures.^[^ ^262^ ^]^ The SE‐*h*TENGs developed by Cong et al. feature a simplified device structure and enhanced practicality for wearable applications with 172 V peak open‐circuit voltage, 94 µA short‐circuit current, and 0.58 mW maximum power output (at 1 MΩ load) based on the sensitivity to various human motion patterns (e.g., finger tapping, clapping, and pounding).^[^ ^262^ ^]^ Wang et al. disclosed a tooth backlash‐inspired comb‐shaped SE‐*h*TENG device that enhances the efficiency of energy harvesting from mechanical vibrations in gear transmissions (e.g., effectively detecting and monitoring gear faults without the need for an external power source).^[^ ^263^ ^]^ The cost‐effective and durable device operated with a peak open‐circuit voltage of up to 7 V, a short‐circuit current of up to 20 nA, and an output power density of approximately 30 µW/m^2^ (at a 100 MΩ load resistance) when tested as a function of different rotational speeds.^[^ ^263^ ^]^ Li et al. revealed a wearable patterned SE‐*h*TENG based on regenerated silk film constructed using ink‐jet printing of Ag inks on the latter that can accurately identify handwritten numbers or letters by transforming the generated output voltage into spectral peaks with spatial identifiers.^[^ ^264^ ^]^ The patterned SE‐*h*TENG has output voltage peaks <20 V and maintains stable performance, allowing for precise digitization and recognition in gamification demonstration without complex algorithms, independent of the absolute amplitude of output signals, sliding speed, and environmental humidity.^[^ ^264^ ^]^ Xiao et al. demonstrated the potential of *h*TENG as an electronic skin for monitoring human body movements, showcasing its application in self‐powered sensing, achieving a peak‐to‐peak open‐circuit voltage of 80 V at a dopant concentration of 2.7 *wt*.% in single‐electrode mode (also presented an alternative contact‐separation configuration).^[^ ^265^ ^]^ They designed *h*TENG in various shapes, including discoid flake, tube, and spiral, to adapt to biomechanical energy harvesting scenarios using flexible poly(vinyl alcohol) hydrogel doped with graphitic carbon nitride.^[^ ^265^ ^]^

**Figure 7:**
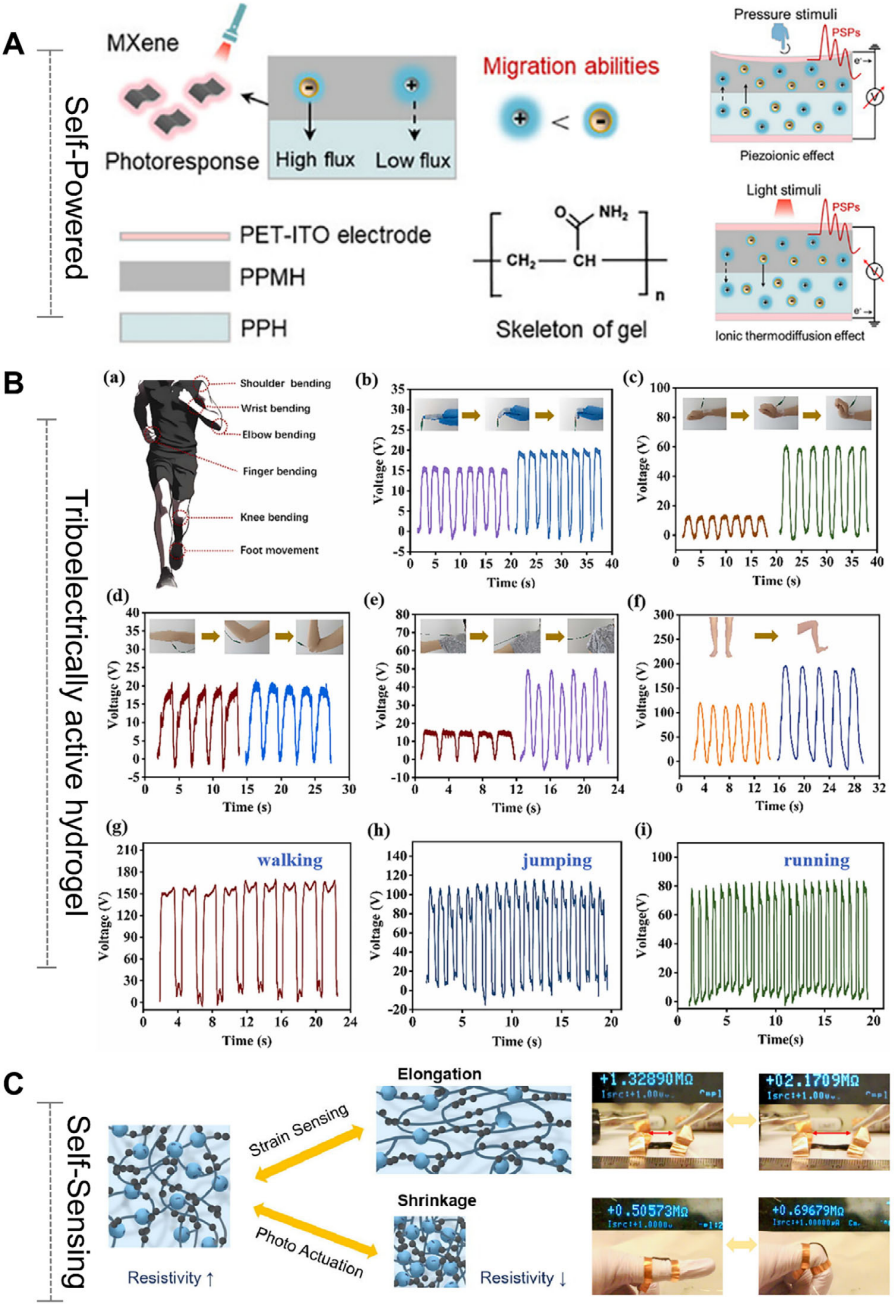
(A) Composition and working mechanism of biomimetic ionic self‐powered hydrogels. Pressure response mechanism: External mechanical stimulation triggers ionic signal generation through the piezoionic effect (top electrode grounded). Reproduced with permission.^[^ ^244^ ^]^ Copyright 2025, Wiley‐VCH. Schematic of the light response. When external NIR light stimulation is applied, ionic electrical signals are generated due to the ionic thermodiffusion effect (with the bottom electrode grounded). (B) Implement self‐powered hTENG sensors in various biological motions such as finger, wrist, elbow, shoulder, knee bending, etc. Reproduced with permission from Ref. [267]. Copyright 2022, Elsevier. (C) Self‐sensing hydrogel actuator: Working principle, real‐time cyclic tensile strain measurement, and curvature sensing on a human index finger. Reproduced with permission from Ref.[288] Copyright 2021, Elsevier. All rights reserved.

Ziyazadeh et al. highlighted the significant advancements in developing dual‐sided (epitomizing potential use on both sides of the patch) and flexible triboelectric nanogenerator‐based hydrogel skin patches, showcasing their potential to revolutionize wound care and promote faster healing.^[^ ^266^ ^]^ Ziyazadeh et al. showed that the energy harvested is sufficient to power small electronic devices and sensors, generating an average open‐circuit voltage of 57 ± 5 V and an average short‐circuit current of 2.2 ± 0.3 µA.^[^ ^266^ ^]^ The triboelectric stimulation using this *h*TENG accelerated wound healing during in vivo rat experiments. It demonstrated a 30% faster wound closure rate compared to control groups, attributed to enhanced cell proliferation and migration, while also highlighting its biocompatibility and non‐toxicity. In another study, Ge et al. synthesized a self‐powered hydrogel using polyvinyl alcohol, polyacrylamide, and zinc sulfate through a one‐step UV‐initiated polymerization process.^[^ ^267^ ^]^ The hydrogels exhibited tensile properties (strain‐to‐failure of 915% and tensile strength of 231 kPa) and electrical properties (open‐circuit voltage of 176 V and a power density of 328 mW/m^2^) (**Figure** **7**B). It was reported that their *h*TENG framework exhibits a highly sensitive response to environmental stimuli and durability, making it suitable for sensing subtle human biomechanical activities such as pulse and limb movements.

#### 2.4.2 Smart Piezoelectric Hydrogels

Piezoelectric hydrogels, characterized by a balance between electroactivity and mechanical compliance, i.e., inherently flexible, have garnered significant attention for biomedical energy harvesting applications.^[^ ^239^, ^240^, ^241^ ^]^ Generally, piezoelectric materials exhibit electromechanical coefficients ranging from 10s to 100s pC/N, enabling them to effectively convert mechanical energy from physiological activities (e.g., heartbeat, muscle contractions, and blood flow) into electrical signals, i.e., highly suitable for developing self‐powered implantable devices and wearable sensors.^[^ ^239^, ^240^, ^241^ ^]^ The successful development of piezoelectric hydrogels, as a subcategory of self‐powered hydrogels, hinges on hybridizing the hydrogel polymer matrix with a piezoelectric phase, where the latter becomes nearly standalone inclusions and does not participate in the physical or chemical crosslinking.^[^ ^240^ ^]^ While perceived as a limitation, this hybridization schema enables preparing a broad range of piezoelectric hydrogels.^[^ ^240^ ^]^ Piezoelectric hydrogels encompass natural biopolymers, such as silk fibroin,^[^ ^268^ ^]^ chitin,^[^ ^269^, ^270^ ^]^ chitosan,^[^ ^271^ ^]^ collagen,^[^ ^272^ ^]^ and bacterial cellulose,^[^ ^273^ ^]^ and synthetic polymers, including polyacrylonitrile,^[^ ^274^, ^275^ ^]^ polyacrylamide,^[^ ^275^, ^276^ ^]^ polyvinyl alcohol,^[^ ^277^ ^]^ poly(ethylene glycol) diacrylate,^[^ ^277^ ^]^ and sodium alginate^[^ ^241^ ^]^ hydrogel matrices and polyvinylidene fluoride (polymer) or barium titanate (ceramic) piezoelectric fillers.^[^ ^240^ ^]^

Vinikoor et al. recently developed an injectable piezoelectric hydrogel comprising short poly‐L‐lactic acid nanofibers and a collagen matrix to activate cartilage healing through self‐powered localized electrical stimulation.^[^ ^278^ ^]^ The authors compared the output voltage of this piezoelectric hydrogel, generating a coherent signal with a consistent amplitude (≈33.7 mVPP) and frequency, while also demonstrating its biocompatibility in both the short‐term (1 to 3 days) and long‐term (14 days).^[^ ^278^ ^]^ Roldan et al. introduced another injectable piezoelectric hydrogel by hybridizing gelatin methacryloyl with piezoelectric barium titanate fillers that are responsive to biomechanical vibrations typical in periodontology, generating ≈10 mV/mm^2^ under clinically relevant 0.9 MPa stress.^[^ ^279^ ^]^ The piezoelectric hydrogel framework is promising for treating periodontal disease while exhibiting antibacterial properties, osteogenic potential, and the ability to reduce inflammation.^[^ ^279^ ^]^ In another study, Wang et al. fabricated transparent and stretchable (800%) piezoelectric hydrogel comprising polyacrylamide and barium titanate nanocubes, which was used as high‐sensitivity pressure sensors detecting forces ranging between 0.25‐6 N and reporting corresponding voltages of 2.5–17 V.^[^ ^276^ ^]^

Li et al. reported a new electroactive hydrogel of polyacrylonitrile‐acrylamide‐styrene sulfate and poly(vinylidene fluoride) with bio‐comparable stretchability (≈380%) and Young's modulus (0.48 ± 0.03 MPa) to human skin for treating pressure injuries/ulcers.^[^ ^275^ ^]^ The open‐circuit voltage measured under 25 to 50 N increased non‐monotonically from ≈20 to 60 mV, respectively, and maintained comparable piezoelectric response after 300 cycles, indicating stability and sensitivity. Wang et al. also developed a self‐powered piezoelectric polyvinyl alcohol hydrogel with polyvinylidene fluoride electroactive polymer phase using a freezing/thawing‐solvent replacement‐annealing‐swelling (to enhance hydrogen bonding within the hydrogel framework) process for diabetic wound repair applications.^[^ ^277^ ^]^ The system achieved a stable and sensitive piezoelectric response with excellent mechanical strength and stretchability (improving the voltage‐acceleration coupling coefficient to 0.432 V/m.s^−2^, compared to merely 0.086 V/m.s^−2^ for neat hydrogel) and good cytocompatibility, significantly promoting proliferation, migration, and secretion of extracellular matrix proteins and growth factors of fibroblasts.^[^ ^277^ ^]^

Lu et al. developed a recyclable pressure sensor using a piezoelectric hydrogel composite of bacterial cellulose and an imidazolium perchlorate molecular ferroelectric, achieving a high sensitivity of 4 mV/kPa over a broad operational range of 0.2 to 31.25 kPa.^[^ ^280^ ^]^ The hydrogels possess electrical and mechanical properties within the biomedical arena, positioning them as ideal material candidates for self‐powered devices for cardiac, pulmonary, skeletal, or visual systems, sensors (static or dynamic pressure, rate, temperature, etc.), promoting healing and growth (e.g., wound dressings, liners for articulating joints, and muscles), and powering implantable smart devices (i.e., power source).^[^ ^239^ ^]^

Although we are advancing toward developing more advanced self‐powered hydrogels for various biomedical applications, specific challenges still create hurdles, such as biodegradability, biosafety, compatibility with internal organs, and utilizing them for powering multiple systems. Before they can be used commercially, further efforts are required to improve their lifespan, compatibility with organs, multi‐powering system, and experiments on large animal models.

### 2.5 Self‐Sensing Actuator Hydrogels

Hydrogels have self‐sensing capabilities by integrating conductive components (*e.g*., ion networks and magnetic particles) to monitor deformation, temperature, or chemical signals in real time while maintaining their inherent driving properties. This integrated design of self‐sensing and self‐actuation greatly expands its application potential in soft robotics, wearables, and biomedical engineering.^[^ ^281^, ^282^ ^]^

One notable development is a self‐sensing bilayer actuator composed of magnetic and ionic conductive hydrogels, which has been evaluated for its actuation and sensing capabilities in soft robotics.^[^ ^283^ ^]^ The bilayer structure comprises a bottom layer of magnetic PAAm hydrogel containing hard magnetic particles of neodymium‐iron‐boron (NdFeB), which enables a rapid response to external magnetic fields and facilitates bending deformation. The top layer was a PAAm hydrogel doped with lithium chloride (LiCl), allowing the sensing of deformation due to ionic conductivity.^[^ ^283^ ^]^ Further advancements include integrating actuation and strain‐sensing capabilities into a homogeneous self‐sensing hydrogel.^[^ ^284^ ^]^ The latter was prepared by mixing a solution of a photo‐crosslinkable poly(N‐isopropylacrylamide) (PNIPAM) hydrogel and polypyrrole (PPy), to mimic the neuromuscular behaviors of living organisms, enabling it to sense motions while simultaneously actuating in response to external stimuli.^[^ ^284^ ^]^ The hydrogel can perform various precise and remotely driven photo‐responsive locomotion tasks, such as signal tracking, bending, weightlifting, object grasping, and transportation, while simultaneously monitoring these motions through real‐time resistance changes. The working principle of this hydrogel self‐inductive actuator is that when the conductive polymer network changes volume in response to external stress or thermal stimulation, its resistivity also changes. It can detect real‐time curvature on the human index finger (Figure 7C).^[^ ^284^ ^]^ The development of a bioinspired multifunctional, self‐sensing, actuated gradient hydrogel designed for remote interaction with soft‐hard robots further illustrates the potential of these materials.^[^ ^285^ ^]^ The gradient structure of self‐sensing hydrogels is created through the rapid precipitation of MoO_2_ nanosheets during the copolymerization of N‐isopropylacrylamide (NIPAM) and sodium alginate (SA).^[^ ^285^ ^]^ The resulting gradient structure integrates ultrafast actuation and high sensitivity, enabling it to mimic the self‐sensing capabilities of natural organisms, such as jellyfish and human tongues. A multifunctional conductive hydrogel with self‐sensing and self‐actuation properties was also developed by incorporating a conductive polymer, polyaniline (PANI), into a double network poly(N‐isopropylacrylamide‐co‐acrylamide)/poly(vinyl alcohol) (PNA/PVA) hydrogel.^[^ ^286^ ^]^ The PNA/PVA/PANI hydrogel exhibited a wide range of tunable mechanical properties and phase transition temperatures. The optimized PNA_12_/PVA_2_/PANI hydrogel showed good elasticity, flexibility (250% strain at ≈26.8 kPa), and stable electrical conductivity. By integrating a layer of optimized hydrogel with a passive PAAm hydrogel layer, the prepared PAAm//PNA_12_/PVA_2_/PANI bilayer hydrogel exhibited rapid and reversible shape deformations in response to thermal stimuli, thereby achieving a thermal‐responsive soft robotic gripper.^[^ ^286^ ^]^ The hydrogel indicated various actuation behaviors, including contraction, bending, light tracking, and weightlifting when exposed to near‐infrared (NIR) light. The potential application of hydrogels was validated by controlling the movements of a hydrogel octopus, highlighting their applicability in soft biomimetic actuating systems.^[^ ^286^ ^]^

Intrinsic anisotropy properties in hydrogels are crucial for enabling them to undergo reversible shape morphing, making them suitable for applications in soft robotics. For example, Li et al. applied a 4D printing technique, direct ink writing (DIW), with smart hydrogel to develop biomimetic anisotropic self‐sensing actuators based on PNIPAM hydrogel containing short carbon fibers (SCFs).^[^ ^287^ ^]^ They created biomaterials that mimic nature's anisotropic and responsive structures, enabling precise control over movement and actuation in response to environmental stimuli, particularly temperature changes. During this process, the SCFs are aligned within the hydrogel matrix under the influence of shear forces as the ink is extruded through a nozzle. This alignment resulted in anisotropic structures that exhibit significant differences in swelling behavior, electrical conductivity, and mechanical properties depending on the orientation of the SCFs.^[^ ^287^ ^]^ The authors successfully created various biomimetic structures, including butterfly‐like actuators, which demonstrate programmable shape morphing and self‐sensing capabilities. Additionally, the self‐sensing hydrogels can detect their own movements through changes in resistance, establishing a feedback loop essential for intelligent robotic applications. However, one notable limitation of that work was the relatively slow actuation rates of the hydrogel actuators, which may hinder their practical applications in fast‐responsive environments.^[^ ^287^ ^]^

Despite their promise, existing self‐sensing hydrogel actuators suffer from compromised sensing‐actuation coupling efficiency and unsatisfactory operational durability. Emerging bio‐fabrication strategies may enable next‐generation systems with enhanced functional integration and reliability.

### 2.6 Self‐Oxygenating Hydrogels

One of the key challenges in tissue engineering and regenerative medicine, tumor therapy, cell transplantation, and organoid culture is ensuring adequate oxygen supply.^[^ ^289^, ^290^ ^]^ Hence, various oxygen‐producing hydrogels have been designed.^[^ ^291^, ^292^ ^]^ According to the oxygen supply mechanism, it can be classified as “Storage‐Release Type/One‐Time Oxygen Supply Type” and “Continuous Self‐Supply Type”.

“Storage‐release type/One‐time oxygen supply type” systems include those that generate oxygen through physical adsorption (e.g., perfluorinated compounds) or chemical decomposition, relying on pre‐loaded substances or limited chemical reactions.^[^ ^293^, ^294^ ^]^ While capable of active oxygen production, these systems lack sustainability due to their finite oxygen reserves. For example, Hassan et al. developed an injectable self‐oxygenating hydrogel composed of silk fibroin (SF) and tyramine‐conjugated alginate (TA‐Alg), which mimics the mechanical environment of the cardiac extracellular matrix to promote repair after myocardial infarction (MI). The core therapeutic strategy relies on two coordinated mechanisms: oxygen‐releasing microparticles (OMPs) encapsulated in the hydrogel react with tissue fluid to continuously generate oxygen, alleviating hypoxia in the infarcted area, while the sustained release of stromal cell‐derived factor‐1α (SDF‐1α) recruits endogenous stem and reparative cells to the injury site. In animal models of myocardial infarction, this combined approach of oxygen supply and cell recruitment demonstrated potent synergistic therapeutic effects, showing great promise for the regenerative treatment of myocardial infarction.^[^ ^295^ ^]^ Additionally, oxygen supply has been demonstrated to promote vascularization and accelerate bone regeneration.^[^ ^296^ ^]^ Using 3D printing technology, researchers developed a bioink composed of polyacrylamide, CaCl_2_‐crosslinked sodium carboxymethyl cellulose, and ZIF‐8‐CaO_2_ nanoparticles. The composite scaffold enables stable and sustained oxygen release through the reaction of water with the CaO_2_ nanoparticles encapsulated in the ZIF‐8 framework. In an in vivo rat cranial defect model, animals treated with this hydrogel exhibited significant new bone formation accompanied by vascular network reconstruction after 12 weeks.^[^ ^296^ ^]^

**Figure 8:**
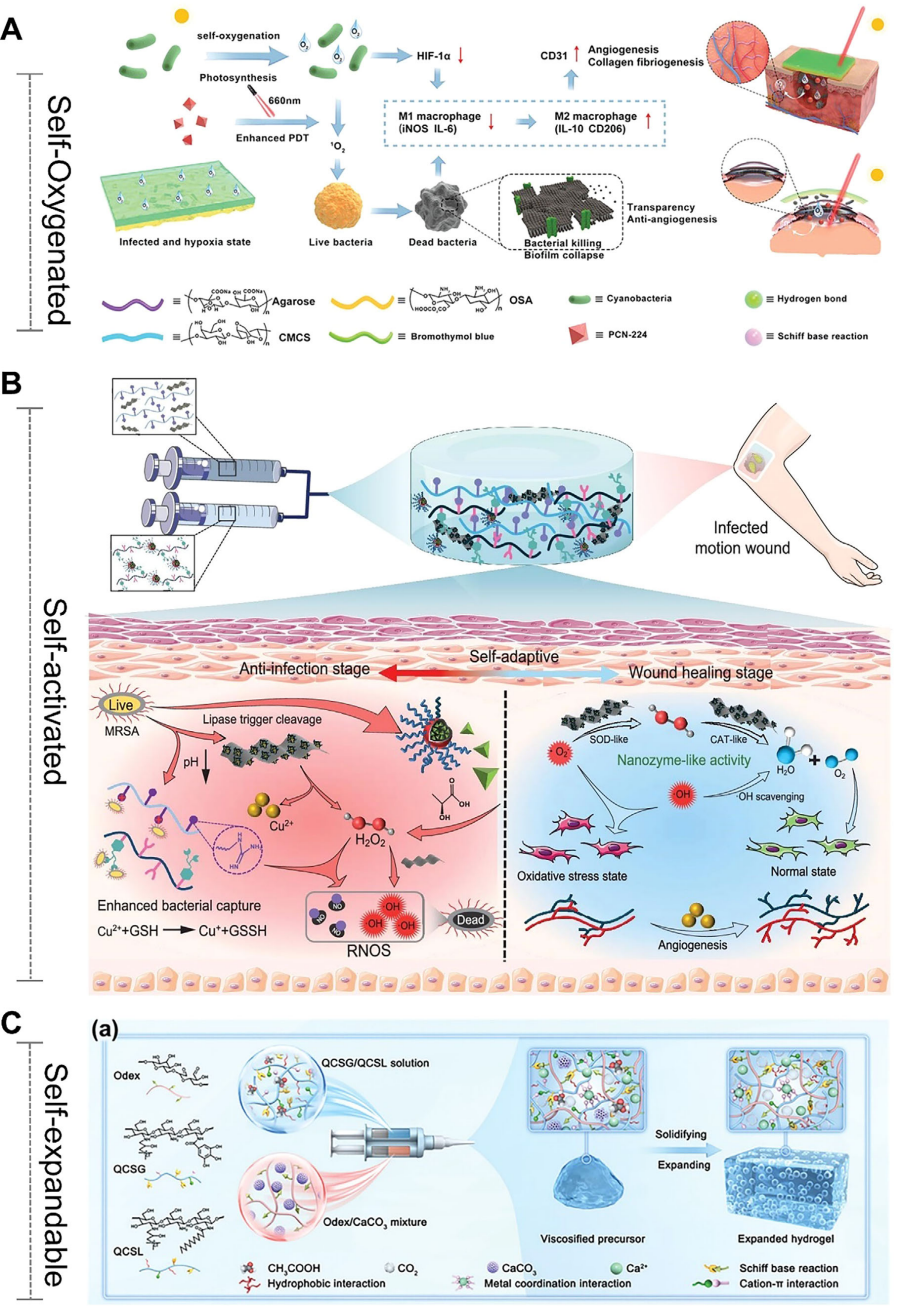
Innovative design and application of self‐oxygenating, self‐activating, and self‐expanding hydrogels. (A) Natural light‐activated self‐oxygenating double‐bilayer hydrogel for diabetic and keratitis wound healing. Reproduced with permission.^[^ ^299^ ^]^ Copyright 2022, Wiley‐VCH. (B) Self‐adaptive therapeutic mechanisms of a bacterial‐responsive, self‐activating antibacterial hydrogel wound dressing with multi‐nanozyme activity for infected sports wound healing. Reproduced with permission.^[^ ^34^ ^]^ Copyright 2024, Oxford University Press (OUP). (C) Schematic illustration of the injectable, self‐expanding, and self‐propelling hydrogel adhesive. Reproduced with permission.^[^ ^305^ ^]^ Copyright 2023, Wiley‐VCH.

“Continuous Self‐Supply Type” can be exemplified by systems utilizing photocatalytic water splitting or microalgae photosynthesis, which achieve long‐term oxygen generation by leveraging external stimuli such as light energy, ensuring sustained self‐sufficiency.^[^ ^245^, ^246^ ^]^ While both storage‐release and continuous self‐supply hydrogels enable programmed oxygen delivery, continuous self‐supply hydrogels are more advanced for clinical applications due to their sustained oxygen generation capabilities. Moreover, their design aligns closely with the concept of programmable hydrogels, offering superior programmability for dynamic hydrogel systems. Consequently, this section emphasizes continuous self‐supply hydrogels, which actively and autonomously generate oxygen in response to environmental stimuli such as light or pH. For example, a novel auto‐oxygen photodynamic therapy (PDT) system using cyanobacteria has been developed based on the photo‐crosslinked GelMA and CeCian‐Cu_5.4_O mixture to treat anaerobic infections and promote tissue repair.^[^ ^297^ ^]^ Cyanobacteria are used as the carrier of photosensitizers, Chlorin e6 (Ce6) and ultra‐small copper oxide nanoparticles (Cu_5.4_O USNPs), which generate reactive oxygen species (ROS) that effectively destroy bacteria and enhance the PDT effect through continuous oxygen production. The study showed that the combination of Ce6, cyanobacteria, and Cu5.4O USNPs had a remarkable bactericidal impact, and almost 100% of anaerobic bacteria were removed under laser irradiation. Moreover, the restriction of an anoxic environment on PDT was significantly reduced due to the oxygen‐producing effect of cyanobacteria. In addition, the system promotes tissue repair by reducing oxidative stress and inflammation, which was improved in an animal (Sprague Dawley rat) model of refractory keratitis and periodontitis. However, one notable limitation of the study is the reliance on laser irradiation to activate the PDT process, which may not be applicable in all clinical settings.^[^ ^298^ ^]^ To further advance the application of self‐oxygenated hydrogels beyond laser‐dependent systems, a double‐layered hydrogel using natural light instead of laser irradiation was developed to address complications associated with refractory diabetic foot ulcers (DFUs) and diabetic refractory keratopathy (DRKs), which are often exacerbated by bacterial infections, chronic inflammation, and inadequate oxygen supply.^[^ ^299^ ^]^ This double‐layer hydrogel with self‐oxygenation function cannot only monitor the infection status but also provide oxygen supply. The inner layer of the hydrogel (alginate and chitosan) incorporated a photodynamic metal‐organic framework (PCN‐224) and a pH indicator (bromothymol blue), and an outer layer (agarose and chitosan) that contained photosynthetic cyanobacteria. The inner layer was designed to respond to changes in pH (during bacterial infection), while the outer layer continuously generates oxygen through photosynthesis when exposed to natural light. The two layers are bonded by Schiff base reaction to ensure a stable interface. The results indicate that the self‐oxygenating double‐layered hydrogel effectively visualized bacterial infections through color changes in response to pH shifts and enhanced the efficiency of PDT via continuous oxygen generation, promoting tissue repair in diabetic wounds in diabetic mice^[^ ^299^ ^]^ (**Figure** **8**A).

In summary, self‐oxygenating hydrogels can activate on‐demand delivery under water or light stimulation, addressing hypoxia in harsh environments such as diabetic wounds, bone tissue engineering, and cardiac injuries. They will also enhance cell activity to improve tissue regeneration and targeted therapies. Future studies should focus on combining oxygenating reagents with other biochemical or biological molecules, such as growth factors, and controlling oxygen supply dynamics to advance tissue engineering.

### 2.7 Other Programmable Hydrogels

Some other advanced programmable hydrogels, engineered to perform specific functions and respond to stimuli in a controlled manner, are particularly used in the biomedical field. This section discusses self‐activated, self‐expandable, self‐destructive, and self‐sensing actuator hydrogels.

#### 2.7.1 Self‐Activated Hydrogels

Self‐activating hydrogels are designed to respond to specific environmental signals (e.g., pH, temperature, or biomolecules), triggering pre‐programmed responses. These responses follow a one‐way, predetermined pathway, manifesting as physicochemical changes without continuous feedback regulation. The design prioritizes the initial triggering mechanism over adaptive modulation in response to subsequent environmental changes.^[^ ^282^ ^]^ One application of self‐activated hydrogels is the release of nitric oxide (NO) through a multi‐enzymatic cascade system. This system is designed to generate glucose, hydrogen peroxide (H_2_O_2_), and NO in response to a slightly acidic environment, such as in *E.coli*‐infected wounds.^[^ ^300^ ^]^ The self‐activated NO release process involves the synthesis of a multi‐enzymatic cascade system utilizing chitosan‐grafted‐polyarginine (CS‐N‐PArg) as the macro‐molecular NO donor and acetylated starch (AcSt‐O‐PAsp) as a glucose donor. Once the cascade system was applied to an *E. coli*‐infected wound (mouse models), the acidic environment triggers the breakdown of the acetal bond in AcSt‐O‐PAsp, releasing starch that glucoamylase (GA) hydrolyses into glucose. GOx then oxidized the glucose to produce H_2_O_2_, which facilitates the oxidation of L‐arginine (L‐Arg) into NO, providing an effective antibacterial treatment for wound healing.^[^ ^300^ ^]^

Self‐activated hydrogels also find applications in self‐healing materials. A healable hydrogel was prepared from a copolymer of N‐isopropylacrylamide and acylhydrazine, P(NIPAM‐co‐AH), crosslinked by polyethylene oxide (PEO) dialdehyde.^[^ ^301^ ^]^ When the hydrogel is damaged, the acylhydrazone exchange reaction can be activated by excess acylhydrazine groups, eliminating the need for any external stimulus. The hydrogel, which contains a significant portion of the PNIPAM segment, exhibited temperature responsiveness at 37 °C, affected by changes in the group ratio. When the temperature is increased, the hydrogel undergoes a phase transition, resulting in changes to its transparency and solubility. Since no in vivo performance data were reported, the mechanical strength and durability of the hydrogel under dynamic biological environments may be a concern.^[^ ^301^ ^]^

Chitosan‐based hydrogels with bacteria‐responsive self‐activating antibacterial properties have been developed for infected wound healing.^[^ ^34^ ^]^ The self‐activating antibacterial hydrogel featured multiple nanozyme activities to enhance the regenerative microenvironment. The hydrogels were fabricated by synthesizing a pH‐sensitive polyethylene glycol‐polycaprolactone‐poly‐β‐aminoester triblock copolymer (PEG‐PCL‐PAE) to create micelles that encapsulate lactate oxidase (Lox). A composite nanozyme (MSCO) was prepared by coating CuO_2_ nanoparticles on MoS_2_ nanosheets. The self‐healing hydrogels are then formed by combining L‐arginine‐modified chitosan (CA) and phenylboronic acid‐modified oxidized dextran (ODP) through dynamic bonds, followed by encapsulating the MSCO and Lox‐loaded micelles within the hydrogel matrix. The antibacterial efficiency increased with decreasing pH, reaching 98.2% against *E. coli* and 99.4% against *methicillin‐resistant Staphylococcus aureus* (*MRSA*) at pH 5.5, reflecting its effectiveness in acidic environments typical of infections. In vivo testing using a mouse infection model further confirmed its antibacterial performance, with the CAOP/M/PL group showing no detectable bacterial colonies (Figure 8B).^[^ ^34^ ^]^

Lastly, self‐activated hydrogels have been explored for artificial skin applications. By integrating graphene and polymers into a thin film, these hydrogels mimic the mechanical self‐healing and pressure sensitivity of natural skin without external power.^[^ ^302^ ^]^ Its self‐healing properties were primarily based on the integration of poly(N, N‐dimethylacrylamide) (PDMAA) and PVA with reduced graphene oxide (rGO). The self‐healing process occurred through mutual diffusion of PDMAA chains and their subsequent hydrogen‐bonding‐driven interactions with each other, allowing the film to recover its electrical and mechanical properties after being damaged. The self‐healing mechanism involves the network reconstruction at the interface due to the mutual diffusion of PDMAA chains, allowing the film to shrink to its initial length and reopen closed pores, recovering its 3D porous structure.^[^ ^302^ ^]^

The future of self‐activated hydrogels is promising for biomedical and tissue regeneration applications. Combining hydrogels with nanotechnology could amplify their effectiveness in medical treatments. Additionally, hydrogels release therapeutic agents, such as nitric oxide, in response to environmental changes, thereby providing a targeted and controlled environment for tissue repair. Self‐activated hydrogels can also mimic natural skin's pressure sensitivity and self‐healing capabilities, enhancing recovery from injuries. More importantly, self‐activated hydrogels activated primarily by temperature present remarkable opportunities for controlled drug release, ensuring that drugs are released only when needed, thereby improving efficacy and reducing side effects. However, despite their potential, large‐scale clinical and environmental validations, especially regarding long‐term performance and biocompatibility, remain essential to ensure their safe, efficient, and sustainable implementation.

#### 2.7.2 Self‐Expandable Hydrogels

Self‐expandable hydrogels are remarkable materials that can undergo controlled volumetric changes, enabling them to adjust to the size and shape of the target tissue or organ. A key distinguishing characteristic of these hydrogels is their exceptional swelling capacity, enabling them to absorb large amounts of fluid and undergo substantial volume expansion. Despite this significant swelling, they maintain their structural integrity during and after the process. This robust property makes them particularly valuable in minimally invasive surgeries, where they can be inserted in a compact form and then expand to fill cavities or spaces.^[^ ^303^, ^304^ ^]^

Injectable self‐expanding hydrogels open new possibilities for creating hemostats for lethal massive bleeding, abdominal organ bleeding, and bleeding from coagulation lesions, presenting a promising solution for critical wound management. For example, Zhao et al. developed an injectable, antibacterial, self‐expanding, and self‐propelling hydrogel adhesive based on oxidized dextran (OD) crosslinked with lauric acid‐modified quaternized chitosan (QCSL) and gallic acid‐modified quaternized chitosan (QCSG).^[^ ^305^ ^]^ The hydrogel also contains porous calcium carbonate microparticles. The function of the hydrogel is to quickly reach deep bleeding sites, adhere to wounds, expand to stop bleeding, and promote hemostasis by rapid gelation. The self‐expanding component of the hydrogel is a foaming reaction between porous calcium carbonate and acetic acid, which provides the self‐expanding and self‐propelling properties to the hydrogel. The optimized hydrogel, containing 30 µL mL^−1^ of acetic acid in hydrogel precursor, exhibited better hemostatic ability compared to commercial combat gauze and gelatin sponge in rat liver and femoral artery bleeding models, rabbit volumetric liver loss massive bleeding models, rabbit abdominal cavity massive bleeding models from invisible bleeding sites, and lethal swine subclavian artery and vein transection noncompressible bleeding models (Figure 8C).^[^ ^305^ ^]^ Additionally, studies have shown the capability of self‐expanding hydrogels in developing advanced stents.^[^ ^304^, ^306^ ^]^

Beyond hemostatic applications, self‐expanding hydrogels have demonstrated potential in the development of advanced stents. A novel self‐expandable biliary stent was developed based on PVA hydrogel to achieve self‐expandability and removability in the bile duct.^[^ ^304^ ^]^ Tube‐shaped PVA hydrogels were prepared and dried under extension. The PVA hydrogel was designed to expand in physiological saline, with a significant increase in inner diameter, allowing it to be used as a self‐expandable stent. The tube‐shaped PVA hydrogels possessed excellent mechanical properties and long‐term dimensional stability. However, the performance of the self‐expanding PVA hydrogels was not evaluated in an animal model in that work.^[^ ^304^ ^]^ Further advancements include the use of self‐expanding PVA with anisotropic swelling behavior in developing a novel stent with endoscopic deliverability for prevalent causes of obstructive jaundice in patients with cholangiocarcinoma, ampullary cancer, and pancreatic cancer.^[^ ^306^ ^]^ The results showed that using a porcine stent model, the tube‐shaped PVA hydrogel could effectively expand the biliary tract without disturbing bile flow in the in vivo experiment. Furthermore, the PVA hydrogels prepared by drying under extension showed structural orientation along the extension axis, leading to anisotropic swelling.^[^ ^306^ ^]^ However, several limitations in that work need to be addressed, including the need for a longer‐term study to assess durability and performance, as well as the investigation of potential biofilm formation over extended periods.

Recently, a novel self‐expanding dried hydrogel (sponge) has been developed for hemostasis applications of incompressible wounds.^[^ ^307^ ^]^ The hemostasis sponge was prepared by combining agarose (AG) and gallic acid‐modified chitosan (GA‐CS) gels through sequential physical mixing, freeze‐drying, and a compression fixation process to create the water‐triggered self‐expanding hemostatic sponge. The cytotoxicity experiment results showed good biocompatibility of different ratios of AG/GA‐CS sponge extracts with L929 cells after incubation for 24 and 48 h. The cell survival rate in all sponge extracts was greater than 96%. The AG/GA‐CS sponges rapidly expanded (greater than 258%) after absorbing Phosphate‐Buffered Saline (PBS). In vivo studies using hemorrhage animal models demonstrated that the AG6/GA‐CS4 group exhibited superior hemostatic effects compared to commercial gelatin sponge. For instance, the total amount of bleeding was sharply reduced by about five times within 5 s in the rat liver defect model. Furthermore, the hemostatic time in the femoral artery hemorrhage model decreased to 3.71 ± 0.80 min when using AG6/GA‐CS4 sponge.^[^ ^307^ ^]^

In conclusion, self‐expandable hydrogels possess great potential in the future due to their ability to swell and conform to tissue shapes while maintaining structural integrity. Their primary application is in minimally invasive surgeries and wound management, including bleeding control, which offers opportunities for broader applications, such as advanced stents and drug delivery systems. This capability for immediate response in life‐threatening situations significantly benefits trauma care. Future studies should focus on performance and safety, particularly in complex clinical environments, ensuring their reliability in sustained treatments.

#### 2.7.3 Self‐destructive hydrogels

In specific applications, hydrogels should degrade or disintegrate after serving their purpose. Self‐destructive hydrogels have been engineered to break down in response to specific triggers, such as enzymatic activity^[^ ^308^ ^]^ or changes in pH.^[^ ^309^ ^]^ These hydrogels offer a controlled degradation process, making them suitable for various biomedical applications. One innovative approach involves self‐destructive hydrogels based on tetra‐PEG (tPEG) crosslinked by bisazide‐modified DNA hairpins. This method enabled the integration of force‐responsive DNA structures into the hydrogel network, allowing the hydrogels to respond to piconewton forces by unfolding the DNA hairpins and exposing cleavage sites for the CRISPR‐Cas12a nuclease. Gold nanoparticles were doped into the hydrogel to visualize and quantify the self‐destruction of the hydrogel. When Cas12a and external force were applied, near‐complete release of the nanoparticles was observed after 3 h of incubation at 37 °C. This demonstrated that the application of mechanical force significantly enhanced the degradation process. Another example is a polyelectrolyte hydrogel, designed for timed‐release properties in self‐rupturing applications.^[^ ^310^ ^]^ The self‐rupturing hydrogels were prepared to exploit the principle of nonuniform cross‐link densities to achieve controlled swelling and subsequent rupture. For instance, the self‐rupturing hydrogels were fabricated by copolymerizing acrylic acid with N, N‐methylenebis(acrylamide) (MBA) as a cross‐linker. The process employed a layer‐by‐layer (LbL) approach to achieve self‐rupturing behavior, utilizing different molar ratios of MBA to acrylic acid to create layers with distinct cross‐link densities. This allowed for the formation of multi‐part gels with non‐uniform cross‐linking. The results indicated that the rupture times for two‐part gels varied significantly based on the cross‐link nonuniformity. The rupture occurred within approximately 1 to 7 days of immersion in swelling media, depending on the specific combinations of MBA: PAA ratios used.^[^ ^310^ ^]^

In cancer therapy, a self‐destructive polymeric carrier was designed to improve the efficacy of chemo‐photodynamic treatment (PDT) for malignant cancers while minimizing off‐target toxicity associated with traditional chemotherapy.^[^ ^311^ ^]^ The self‐destructive polymeric nanoparticles incorporated a ROS‐sensitive thioketal linkage that allowed the polymer to degrade in response to ROS generated during PDT. The self‐destructive hydrogels' fabrication method involves synthesizing a ROS‐responsive polymer through post‐polymerization modification. The thioketal linkages were grafted onto the aliphatic polycarbonate (APC) pendant chains to create Polyethylene Glycol‐Poly(butylene carbonate)‐Thioketal (PEG‐PBC‐TK). Doxorubicin (DOX) was then conjugated to this polymer via the thioketal bond, forming PEG‐PBC‐TKDOX micelles, the self‐destructive carrier hydrogel. The self‐destructing polymer nanoparticles encapsulated DOX and Ce6 with drug loading efficiencies of 32.2% and over 95%, respectively. The nanoparticles demonstrated a 70.6% reduction in molecular weight of PEG‐PBC‐TK within 1 h and drug release upon exposure to ROS, confirming the effectiveness of the thioketal linkages in facilitating drug release. Through enhanced accumulation and penetration, PEG‐PBC‐TKDOX micelles promoted tumor‐targeted drug delivery and therapy in oral cancer mice. That study highlighted the capability of PEG‐based self‐destructive polymeric carriers to efficiently co‐deliver a chemotherapeutic agent, DOX, and Ce6.^[^ ^311^ ^]^

Self‐destructing hydrogels show great potential in targeted delivery and local therapy due to their precise responsiveness (ROS, enzyme, or pH stimulation), efficient drug delivery, and controlled release. However, technology still faces key challenges such as precise control of degradation rate, optimization of biocompatibility, and stability of the large‐scale preparation process, which should be the research focus in the future.

## 3 Maturity of Programmable Hydrogel Technology

Programmable hydrogels are explicitly defined as materials able to change their properties and functions periodically, reversibly, and/or sequentially on demand.^[^ ^312^ ^]^ This distinguishes them from other responsive hydrogels whose changes might be passive or irreversible. Their maturity can be understood across three key levels:

### 3.1 In‐Vitro Studies: High Maturity

Programmable hydrogels demonstrate a high level of maturity in in vitro studies. At this stage, researchers meticulously synthesize and characterize these materials in controlled laboratory environments. This includes designing hydrogels that precisely control their physical properties and chemical functions. For example, photo‐mediated strategies can be used for dynamic modulation of biophysical cues within hydrogels, such as matrix mechanics, which in turn can tune the behavior of encapsulated cells.^[^ ^313^ ^]^ Significant advancements have been made to transform simple hydrogels into responsive systems based on Boolean logic, gating decisions by incorporating functional peptides and proteins.^[^ ^314^ ^]^ The current state of in‐vitro research allows for precise control of hydrogels with user‐controlled means to match specific cues for mechanobiology studies. These systems can respond to various stimuli (e.g., pH, temperature, light, electricity, and magnetic fields) for purposes like biosensing, targeted drug delivery, and tissue engineering.^[^ ^315^ ^]^ Some studies are even exploring the creation of programmable systems based on hydrophilic polymers capable of recording executable programs, akin to microcontrollers.^[^ ^316^ ^]^ This foundational in‐vitro work forms a robust basis for further translational efforts.

### 3.2 In‐Vivo Studies: Growing Maturity, Varied Success

The maturity of programmable hydrogels in pre‐clinical (in vivo) studies is growing, with numerous applications demonstrating therapeutic potential in animal models. These studies are crucial for bridging the gap between controlled laboratory findings and complex biological systems. Programmable biotechnologies are leading to smart injectable materials with potential.^[^ ^314^ ^]^ Injectable hydrogels, as a broad category that includes many programmable systems, are widely investigated for drug delivery and tissue engineering applications.^[^ ^317^ ^]^ In‐vivo biocompatibility assessments of hydrogels are routinely conducted through subcutaneous implantation in animal models, with studies showing excellent biocompatibility and no significant toxic reactions in major organs.^[^ ^318^, ^319^, ^320^ ^]^ For example, ultra‐durable cell‐free bioactive hydrogels have shown good biocompatibility and on‐demand drug release for cartilage regeneration.^[^ ^320^ ^]^ Photothermal‐sensitive nanocomposite hydrogels have demonstrated excellent biocompatibility in animal models.^[^ ^319^ ^]^ However, despite promising results, challenges persist. While hydrogels have shown promising therapeutic effects in vitro, their long‐term effectiveness in vivo is still largely unspecified due to the complex cellular microenvironment.^[^ ^321^, ^322^ ^]^ Unexpected side effects and complications can arise, highlighting the need for extensive in vivo evaluation.^[^ ^321^ ^]^

### 3.3 Clinical Studies: Nascent Maturity

The clinical translation of programmable hydrogels currently remains at a nascent stage of maturity. Despite extensive in vitro and in vivo research, their widespread application in clinical settings is limited. While many scientific reports have been published on smart hydrogels, which encompass programmable hydrogels, a relatively low number of these products are currently being used in clinical trials.^[^ ^323^ ^]^ The transition from preclinical academic research to broad clinical use is notably challenging, time‐consuming, and expensive.^[^ ^324^ ^]^ Although certain hydrogel technologies have secured regulatory approval for specific healthcare applications, such as cancer treatment or aesthetic corrections,^[^ ^317^ ^]^ and some hydrogel‐based repair materials have entered clinical trials (e.g., for cartilage defects),^[^ ^312^ ^]^ the broader adoption in clinical practice is hampered by stringent requirements for safety, efficacy, and reproducibility in human applications. Challenges include ensuring sensitivity and specificity to minimize non‐specific responses, guaranteeing biocompatibility without adverse effects, and ensuring effectiveness while minimizing interactions between multiple functions. The more sophisticated a material is designed to be, the more difficult it is to attain clinical translation.^[^ ^325^ ^]^ More comprehensive studies are needed to fully understand the long‐term biological responses and signaling transductions within programmable hydrogels, which are essential for expanding their applications beyond limited drug delivery.^[^ ^321^, ^322^ ^]^

## 4 Fundamental Regulatory Challenges

**Figure 9:**
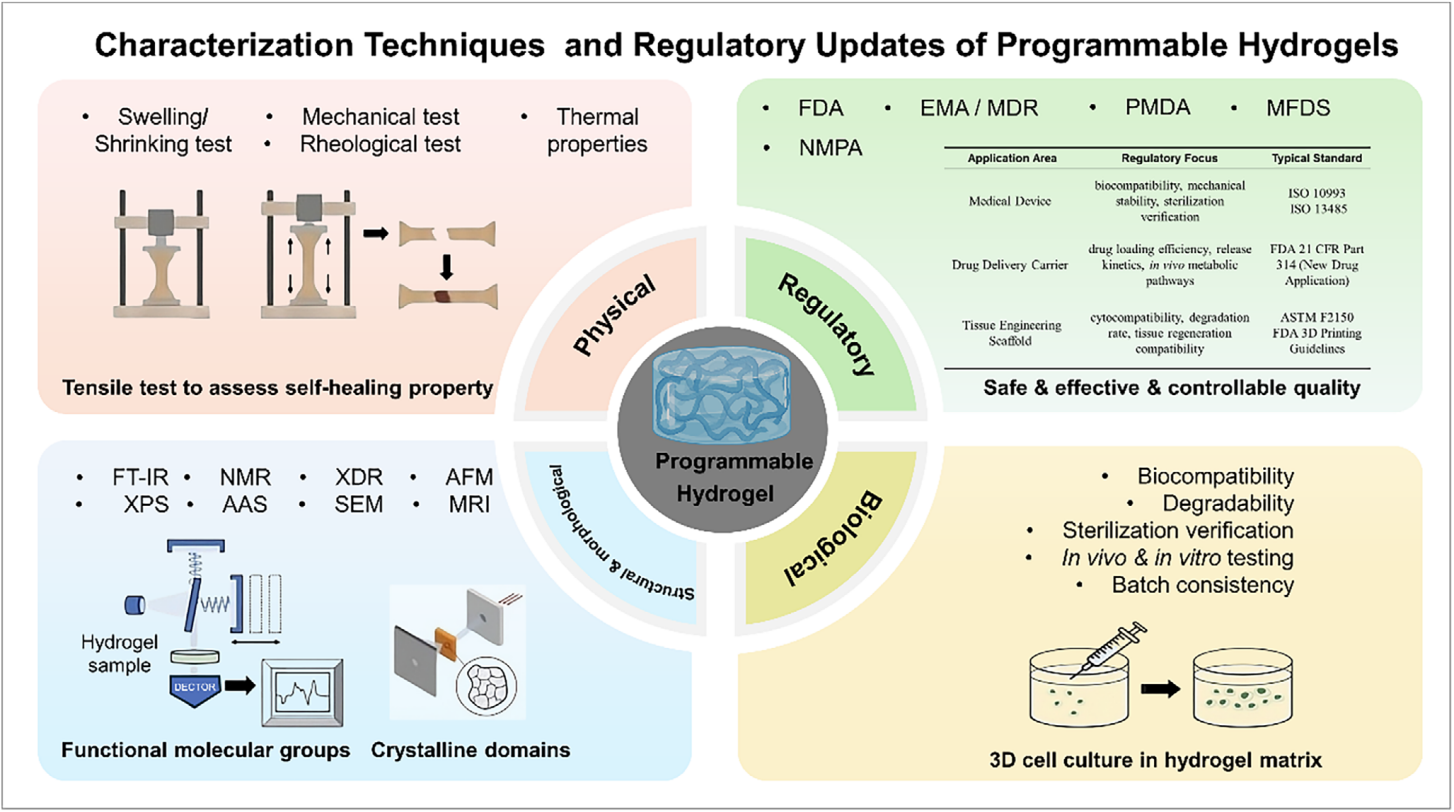
Multiscale characterization techniques of programmable hydrogels and clinical regulations (Created with BioRender.com).

Research on biomedical applications of programmable hydrogels has grown exponentially recently, from 1000 publications in 2006 to more than 7350 in 2021.^[^ ^326^ ^]^ However, fabricating a hydrogel for biomedical applications remains challenging due to strict medical regulations; comprehensive characterization of the structure, behavior, and performance of the programmable hydrogel is required to facilitate its clinical regulatory process (**Figure** **9**). Programmable hydrogels are mainly used in the medical field as medical devices and drug delivery systems, and their clinical applications are strictly regulated. The European Medicines Agency (EMA) and the U.S. Food and Drug Administration (FDA) are the main regulatory agencies. EMA and FDA classify medical devices into three types. Class I medical devices include low‐risk devices for users and do not require prior authorization. Class II are moderate‐risk medical devices, and they are used to treat organs and tissues without penetrating deeply. Class III medical devices are high‐risk because of their significant interactions with the body and require Premarket Approval (PMA).

A rigorous process to accept a medical device usually takes five years. However, if the hydrogel delivers drugs or cells, the device is in the advanced therapy medicinal products (ATMPs) category, and the approval time takes 7–10 years. According to the FDA, regular hydrogels are classified in class I according to the Code of Federal Regulations, Title 21, Volume 8, Chapter 1, Part 878, Subpart E, Sec. 878.4022. Simple hydrogels for wound or burn dressing are only classified as class I,^[^ ^327^ ^]^ and programmable hydrogels are classified as class II and class III. The Medical Device Regulation (MDR) in Europe considers a programmable hydrogel a class III device, mainly due to long‐term contact with tissue and possible biological effects due to its intent to administer or remove medicinal products from the body.^[^ ^328^ ^]^ This means detailed documentation on safety and efficacy, and a premarket clinical investigation is necessary.^[^ ^329^ ^]^ Programmable hydrogels classified as class III are high‐risk medical devices, so preclinical tests are obligatory. The stringent and time‐consuming regulatory requirements, such as lengthy approval timelines (averaging 5–7 years), ambiguous classification standards for dynamic materials (*e*.g., between medical devices and drugs), the absence of Good Manufacturing Practice (GMP) standards for in‐situ formed hydrogels, and the need for long‐term degradation data (tracking over 5 years), significantly impact both the product translation process and the early‐stage research and development of programmable hydrogels. These challenges create a bidirectional feedback loop with research.

To address these challenges, researchers generally adopt a “design for regulation” strategy, which mainly includes: Regulatory constraints shape laboratory research by encouraging a “design for regulation” approach. This includes adopting modular designs that separate functional components (*e.g*., sensing, response, and treatment modules), prioritizing single stimulus‐response mechanisms (*e.g*., pH‐triggered systems), and selecting pre‐approved base materials (*e.g*., polyethylene glycol derivatives) to mitigate regulatory risks from the outset. Regulatory agencies, such as the FDA, through its “Breakthrough Device” program, provide pathways to support innovative technologies with significant clinical potential, aiming to balance safety with innovation. This dynamic interplay underscores that the future of programmable hydrogel research depends not only on technical advancements but also on alignment with regulatory science principles.

Over the last 40 years, more than 100 hydrogel products for tissue regeneration, tissue augmentation, facial correction, contact lenses and other ocular applications, wound dressing, and drug delivery devices have been approved by both the FDA and EMA (Figure 9). This number rose in the last few years.^[^ ^330^ ^]^ For example, Apligraf, a fibroblast and keratinocyte‐induced collagen hydrogel, is an efficient tool for diabetic foot ulcer and venous leg ulcer treatment and was approved by the FDA in 2000. While the use of hydrogels in the industry is well funded, programmable hydrogels have an even more recent introduction to clinical use. The main area of commercialization for these types of hydrogels focuses on drug delivery. Only a few cases have entered clinical use,^[^ ^331^ ^]^ including Jelmyto, a smart hydrogel made from Pluronic F‐127, PEG‐400, and hydroxypropyl methylcellulose (HPMC), currently used for mitomycin delivery and approved by the FDA in April 2020.^[^ ^330^ ^]^ Other hydrogel products with a longer commercial time can be classified as smart hydrogels^[^ ^332^ ^]^ due to their controlled‐release, such as ATRIDOX, a subgingival controlled‐release product approved by the FDA in 1998 and composed of a two‐syringe mixing system that, upon contact with the crevicular fluid, the liquid product solidifies and then allows for controlled release of drug for 7 days.^[^ ^333^ ^]^

Programmable systems that perform active, long‐term functions are more likely to be treated as higher‐risk combination products, substantially complicating the regulatory path. For instance, in the future, programmable hydrogels will likely focus more on gene‐loaded hydrogels with integrated sensors for gene abnormalities treatment and pathogen‐responsive hydrogels for local infection treatments.^[^ ^331^ ^]^ These systems are complex and multiply the possible interactions and safety questions. For these reasons, they would be placed under overlapping regulatory regimes (device, biologic, or ATMP). Furthermore, concerns about off‐target effects, genetic containment, and long‐term monitoring would be raised. Longer, more conservative review and stronger demands for reproducible, mechanistic evidence would be necessary before granting broad clinical approval. These challenges will shape the research topics. For instance, researchers can conduct more robust quantitative characterizations of dynamic performance (e.g., trigger thresholds and response kinetics) alongside traditional biocompatibility tests. Teams working with biological or gene‐based modules increasingly build in genetic safety measures (high‐fidelity gene editors, kill switches, or containment strategies), anticipating the kinds of data that would be required under ATMP or biologics guidance. Furthermore, the use of simulation tools would increase to consider different scenarios and mechanisms. By treating regulatory expectations as design constraints, current research on programmable hydrogels is adapting to generate the predictable, well‐characterized, and manufacturable systems that regulators require.

### 4.1 Challenges in Clinical Translation and Stability

A significant hurdle for smart hydrogels in clinical applications is ensuring their sustained functionality and predictable stability within the complex physiological environment. Despite extensive research and numerous publications on stimuli‐responsive hydrogel systems, only a limited number have successfully transitioned to practical clinical use.^[^ ^334^ ^]^ The majority of these systems prove unsuitable for product development due to various limitations, including insufficient and imprecise stimulus responses, efficacy in human clinical trials, and biodegradation rate control. There is a critical need for novel smart hydrogel systems that exhibit enhanced and precise stimuli responses in clinical trials. Many proposed systems, such as glucose‐responsive hydrogels, demonstrate sluggish responsiveness to fluctuations in biological parameters, such as blood‐glucose levels.^[^ ^334^ ^]^ This lack of precise and timely response hinders their clinical utility. While some smart hydrogels have shown promising outcomes in animal models (e.g., A1 and B29‐oligofucosyl‐insulin in diabetic dog and minipig models), successful evaluation of these systems in human clinical trials remains unachieved.^[^ ^334^ ^]^ This discrepancy is likely due to an incomplete understanding of quantitative differences across species, complicating the prediction of human clinical outcomes. Precisely controlling the biodegradation rate is crucial. If a hydrogel degrades too slowly, it can impede new tissue growth and lead to transplant failure. Conversely, overly fast degradation compromises structural integrity and therapeutic agent delivery.^[^ ^11^ ^]^ Research continues to focus on customizing degradation rates for controlled release of therapeutic agents.^[^ ^11^ ^]^ An ongoing challenge is enhancing the hydrogels' capacity to adapt to the body's dynamic functional and pathological conditions, ensuring they remain resilient and efficient in biological settings.

### 4.2 Biocompatibility of Novel Chemistries and Potential Risks

The integration of novel chemistries and functionalized components in smart hydrogel development raises significant biocompatibility and safety concerns. With the increasing use of newly synthesized polymers and chemical components, it is imperative to thoroughly assess and verify their safety before clinical application. Materials approved by the FDA or those with a prolonged history of safe use without notable side effects are preferred choices for fabricating stimuli‐responsive hydrogels.^[^ ^334^ ^]^ Microstructures, external hybridization, and potential immunological reactions remain obstacles in the production and application of smart hydrogels.^[^ ^11^ ^]^ Future research needs to focus on developing hydrogels capable of regulating and suppressing the body's immune responses. In addition, solvents used during the manufacturing of smart/stimuli‐responsive hydrogels can pose a threat of toxicity, necessitating further investigation into polymer amalgamation during crosslinking to mitigate these risks. Despite advancements, extensive studies are still required to confirm the safety and efficacy of programmable hydrogels before widespread clinical application. The translation from preclinical academic research to clinical use is inherently challenging, time‐consuming, and expensive, often preventing promising technologies from reaching the market.^[^ ^16^ ^]^

### 4.3 Advanced Control and Functional Challenges

Beyond fundamental safety and efficacy, programmable hydrogels encounter specific challenges in achieving sophisticated control and predictable functional changes within the body. Ideally, programmable hydrogels would allow for precise spatiotemporal control of changes. However, most currently developed programmable hydrogels undergo macroscopic changes, affecting the entire bulk of the material.^[^ ^334^ ^]^ While high‐focus stimuli like light (*e.g*., two‐photon techniques) offer high spatial resolution, applying such precise stimuli for real‐world applications (e.g., protein delivery) in a three‐dimensional space is challenging. It is particularly difficult to differentiate the extracellular space from randomly distributed cells within a hydrogel in vitro or tissue in vivo. Therefore, achieving effective spatiotemporal control remains a significant challenge. Integrating multiple external and internal triggering events for a single functional change offers a potential solution, but further research is needed to enable responsive behaviors to coherent or sequential stimuli at both macroscopic and microscopic levels, ensuring local changes do not significantly affect overall hydrogel properties.^[^ ^334^ ^]^ Functional changes within hydrogels are frequently interconnected. For instance, altering the pore size of a hydrogel can simultaneously impact its permeability, mechanical properties, and ligand density. These changes, in turn, can affect cell attachment and protein release. The ability to decouple these simultaneous changes is crucial for a deeper understanding of cellular behavior and for developing more effective therapeutic strategies.^[^ ^334^ ^]^ The effectiveness of functional changes in programmable hydrogels often exhibits a non‐linear relationship with time and tends to decay with repeated stimulation. This phenomenon occurs because changes in the hydrogel are often concentration‐dependent. For example, the amount of protein released decreases with each pulsatile cycle as the concentration gradient diminishes.^[^ ^334^ ^]^ This issue also applies to other functional changes, such as the increase or decrease of stiffness. These concerns with functional decay must be carefully considered during the design of programmable hydrogels for biomedical applications, especially those requiring frequent and periodic changes, such as continuous insulin release for diabetes management.^[^ ^334^ ^]^

## 5 Conclusion, Challenges, And Prospects

### 5.1 Conclusion

Programmable hydrogels represent a groundbreaking advance in intelligent material sciences, offering highly adaptable platforms that respond to various environmental stimuli. Their primary strength lies in their broad versatility, allowing tailored functionalities for applications such as drug delivery, tissue engineering, and wound healing. These innovative materials are engineered to undergo well‐defined transformations, from releasing therapeutic agents and expanding to fill tissue voids, to degrading or altering their structure in response to specific physiological cues like changes in pH, temperature, or the presence of biomolecules. Within this landscape, recent developments have introduced innovative hydrogel types such as self‐activated and self‐oxygenated hydrogels, which can respond to physiological triggers or address tissue hypoxia by generating oxygen, thus significantly supporting healing processes. At the same time, self‐expandable hydrogels have been designed to modulate their volume according to the needs of minimally invasive interventions, adjusting seamlessly to the targeted tissue environment and enhancing their effectiveness as hemostatic agents. The mechanisms driving these changes are rooted in sophisticated molecular interactions, with enzymatic reactions or chemical modifications frequently serving as internal triggers. Additionally, the recent integration of self‐powered hydrogel systems, such as those exploiting triboelectric or piezoelectric principles, opens new possibilities for powering sensors, actuators, and micro‐robotic biomedical devices. These innovations underscore the immense potential of programmable hydrogels as the foundation for next‐generation medical technologies, as evidenced by promising results from preclinical studies and device‐level demonstrations.

### 5.2 Current Challenges

Despite this remarkable progress, several significant challenges remain to be addressed before programmable hydrogels can achieve widespread clinical adoption. One of the key obstacles involves the inherent complexity of multi‐component hydrogel systems, which, while providing diverse functionalities, often complicate reproducibility and increase the likelihood of adverse immune reactions. Achieving a simple, yet multifunctional, design that incorporates essential properties while ensuring safety and consistency continues to demand a nuanced understanding of biological interactions and material science. Moreover, the need for application‐specific customization adds another layer of complexity, as each clinical application may require a distinct set of material characteristics. Structural stability is crucial: some clinical scenarios necessitate hydrogels that maintain their integrity over extended periods, while others demand rapid and complete degradation without leaving residual components behind. Meeting these divergent requirements calls for careful material selection and design at the molecular level. Another largely unexplored frontier is the development of dynamic hydrogels capable of effectively transporting hydrophobic drugs, which holds promise for broadening the therapeutic scope of hydrogel‐based treatments but remains limited by solubility and bioavailability issues. In addition, there are ongoing concerns regarding these materials' long‐term biocompatibility, toxicity, and safety when introduced into the human body. This area currently lacks comprehensive clinical data. Finally, challenges associated with the scalability and reproducibility of hydrogel manufacturing processes continue to hinder large‐scale production and quality assurance, ultimately impacting the transition from laboratory research to practical, real‐world medical solutions.

### 5.3 Future Outlook

Future research should focus on leveraging interdisciplinary technologies such as artificial intelligence, flexible electronics, and synthetic biology to develop next‐generation programmable hydrogels that combine high efficiency, quantifiable performance, and clinical applicability. These advanced materials should exhibit dynamic closed‐loop feedback, biomimetic synergy, and strong clinical relevance, enabling intelligent real‐time responses and seamless integration with biological systems. To realize the full potential of programmable hydrogels, clearly defined, measurable objectives and interdisciplinary technological integration are essential. Key areas for innovation include hydrophobic drug delivery, self‐powered systems, and scalable, clinically oriented manufacturing.^[^ ^335^ ^]^ Short‐term goals include leveraging generative AI to reduce hydrogel development cycles by 50%^[^ ^336^, ^337^ ^]^ and employing machine learning to enhance hydrophobic drug loading and release efficiency to over 80%.^[^ ^338^ ^]^ Long‐term objectives focus on developing intelligent systems and highly controllable production processes, including designing smart hydrogels with closed‐loop feedback capable of responding to physiological signals within 15 min,^[^ ^339^ ^]^ and utilizing digital twin technology to virtually optimize in‐situ forming and manufacturing processes, achieving batch‐to‐batch variability to within ±5%.^[^ ^336^ ^]^ Additionally, integrating flexible electronics enables self‐powered monitoring, while synthetic biology approaches facilitate the development of hydrogels with on‐demand degradation profiles.^[^ ^340^, ^341^ ^]^ Furthermore, AI‐driven regulatory evaluation models, informed by real‐world data (RWE), can streamline compliance processes.^[^ ^342^ ^]^

The use of programmable matrices in organoid technology is another promising area that is rapidly evolving, driven by interdisciplinary approaches. Organoids, which are miniature in vitro cell culture models mimicking organ characteristics, are powerful tools for studying human development and disease.^[^ ^343^ ^]^ Historically, organoid culture has relied on undefined and variable matrices like Matrigel, which poses limitations for scalability, reproducibility, and clinical translation.^[^ ^344^, ^345^ ^]^ Programmable hydrogel matrices offer a superior alternative by providing defined and tunable microenvironments to guide organoid growth and development.^[^ ^45^, ^346^, ^347^ ^]^ There are several advantages and applications of programmable hydrogels in organoid technology. For instance, synthetic hydrogels can be precisely tuned in terms of their physical properties (e.g., stiffness, porosity, elasticity) and biochemical cues (e.g., cell‐binding ligands).^[^ ^346^, ^347^ ^]^ This allows for the creation of rigorously defined microenvironments that mimic the native extracellular matrix, crucial for guiding cell behavior and organoid morphogenesis. These advancements aim to bridge the gap between multifunctional material design and personalized medical applications, transforming programmable hydrogels into clinically viable solutions for tailored treatments.

## Conflict of Interest

The authors declare no conflict of interest.

## Data Availability

The data described in the article are available at https://zenodo.org/uploads/17098951. We would appreciate if other researchers could benefit from our literature and results. This will foster discussions and collaboration among scientists worldwide
